# Modern broiler chickens exhibit a differential gastrointestinal immune and metabolic response to repeated CpG injection relative to a 1950s heritage broiler breed

**DOI:** 10.3389/fphys.2024.1473202

**Published:** 2024-11-01

**Authors:** Bridget A. Aylward, Casey N. Johnson, Famatta Perry, Rose Whelan, Ryan J. Arsenault

**Affiliations:** ^1^ Department of Animal and Food Sciences, University of Delaware, Newark, DE, United States; ^2^ Food and Feed Safety Research Unit, Southern Plains Agricultural Research Center, United States Department of Agriculture, Agricultural Research Service, College Station, TX, United States; ^3^ Evonik Operations GmbH, Birmingham, United Kingdom

**Keywords:** broiler, chicken, immune, CpG, metabolic

## Abstract

The Athens Canadian Random Bred (ACRB) heritage broiler breed, which has not been selectively bred since the 1950s, is a point of comparison to the modern-day broiler and could highlight potential genetic-derived differences in immune responses. To observe the modern and heritage birds’ immune responses in action, the innate immune ligand CpG oligonucleotides were administered at multiple time points through the birds’ lives from the day after hatch to day 35 post-hatch. This study allowed for the observation of changes in metabolic and immune signaling in response to repeated injections of a known Toll-like receptor (TLR) ligand, CpG. Jejunum and cecal tonsil samples at multiple time points during grow out were collected and used for kinome array analysis to measure kinase activity in immunometabolic signaling pathways in the gut tissue. In addition cytokine gene expression was measured in these tissues. The modern birds’ response to the treatment was more innate and showed evidence of metabolic energy shift. The heritage birds’ response to the treatment was adaptive, with metabolic changes indicative of a well-regulated response. Overall, the results from this study suggest that modern broiler chickens do not adequately balance resources between growth and immune responses during an immune challenge, and this deficit is most evident around the 2-week post-hatch time point. This is a critical time for these birds, as their muscle deposition continues to accelerate, and they are vulnerable to disease challenges. Ideally, future work can clarify the reason for this response discrepancy in the modern broiler and therapeutic interventions to rescue this phenotype could be elucidated.

## 1 Introduction

The modern broiler chicken is the product of over 60 years of highly selective breeding based predominantly on production parameters. These birds have an extremely rapid rate of growth, particularly with respect to their breast muscle tissue, and are highly feed efficient. These attributes have contributed to the expansion of the poultry industry into one of the largest animal production industries in the United States, with an estimated value of 31.7 billion dollars in 2018 (“Poultry - Production and Value 2018 Summary 05/23/2019,” 2018). Due to these phenomenal advancements in poultry genetics, chicken is the most popular meat consumed globally (Whitton et al., 2021). In addition to accelerated growth, however, the birds experience skeletal, cardiovascular, metabolic and immune issues, possibly as a byproduct of selectively breeding for growth (Cheema et al., 2003; Olkowski, 2007; Scheele, 1997). Due to the removal of growth promoting antibiotics from poultry feed formulations, broiler chickens are now left susceptible to disease challenges such as necrotic enteritis that the poultry industry has not had to contend with in the last 5 decades (Lanckriet et al., 2010; Kaldhusdal et al., 2016). Furthermore, the removal of antimicrobials from poultry production presents a human health and safety concern, as broiler chickens do not mount sufficient immune responses to clear *Salmonella* infections that may be reduced by antimicrobial administration (Abudabos et al., 2016; Vermeulen et al., 2017). It is necessary to understand and address the modern broilers’ immune response to disease challenges from both an animal welfare standpoint as well as a human food safety standpoint.

To place the modern broiler’s immune response in context, heritage broiler breeds provide a standard to which the modern birds can be compared. Heritage broiler breeds are commercial broiler production birds that have not undergone continued selective breeding for performance parameters. In this study, we use the Athens Canadian Random Bred (ACRB) heritage broiler breed, a pedigreed controlled commercial breed which has been managed at the University of Georgia to maintain the genetics comparable to those of 1958 (Hess, 1962; Collins et al., 2016). ACRB birds show more robust antibody immune responses than modern broilers, whereas modern broilers demonstrate cell-mediated immune responses (Cheema et al., 2003). ACRB birds are also better able to cope with stressors such as heat stress (Berrong and Washburn, 1998). Growth rate and body weight have been shown to impact immune parameters such as antibody responses, therefore it is interesting to observe and compare immune responses in a fast-growing and a slow-growing broiler (Yunis et al., 2000; 2002).

To study the differences in immune response between the heritage broiler and its modern counterpart, it is informative to observe the response to a known immunostimulatory treatment. CpG oligonucleotides (ODN) are synthetic oligonucleotides that induce the immunostimulatory effects of bacterial DNA by binding to the avian Toll-like receptor (TLR) 21 and initiating downstream signaling (Hemmi et al., 2000; Latz et al., 2004; Brownlie et al., 2009; Aylward et al., 2022). *In vitro*, CpG ODNs have been shown to cause increased mRNA expression of proinflammatory cytokines such as interleukin (IL)1β and interferon (IFN) γ and nitric oxide production in HD-11’s, a chicken macrophage cell line (He et al., 2003). Furthermore, chickens and mice that are treated with CpG and then given a bacterial challenge, such as *Listeria monocytogenes* or *Salmonella* species are protected from infections and morbidity associated with these bacterial species (Krieg et al., 1998; Klinman et al., 1999; He et al., 2005). In the current study, modern and ACRB broilers were injected with CpG ODNs at multiple time points throughout the experimental period, with the first injection administered the day after hatch. Tissue samples were collected prior to and 24-h post-injection to track each broiler’s response to the immunostimulant after repeated exposures.

To characterize the birds’ responses to the CpG treatment, kinome arrays were utilized to assess kinase activity along immunometabolic signaling pathways (Johnson, et al., 2023). Kinases’ phosphorylation of target proteins has a direct impact on downstream activation or inhibition of immune and metabolic signaling pathways. Kinome arrays provide a real-time snapshot of the function of kinases active in the tissue of interest. Kinome analysis of the cecal tonsil and jejunum at different timepoints throughout the grow-out period provide a picture of the birds’gut-associated immune and metabolic response to the repeated CpG treatment. qPCR was also performed to assess the mRNA expression of key inflammatory cytokines that are known to show altered expression after stimulation with CpG.

## 2 Materials and methods

### 2.1 Bird husbandry

Embryonic, day 16 post-lay Ross 308 eggs were acquired from Pedigree Chicks (Beaver Creek, PA) and were incubated in Jamesway incubators (Jamesway Incubator Co. Inc., Cambridge, ON, Canada) at 37.5°C and 60% humidity until day of hatch. Newly fertilized Athens Canadian Random Bred (ACRB) eggs were shipped from the University of Georgia Poultry Science Department (Athens, GA). These eggs were incubated in the same incubator conditions until day of hatch. On day of hatch chicks were removed from the incubators when dry and placed into one of eight colony houses on the University of Delaware campus farm (Newark, DE). The houses were equipped with clean pine shavings, bell waterers, and chick feeders, and the temperature was maintained at approximately 35°C for the first week of life. Feed and water were provided *ad libitum*. Birds were changed from starter feed to grower feed on day 12 and from grower feed to finisher feed on day 25.

### 2.2 Experimental design

**FIGURE 1:**
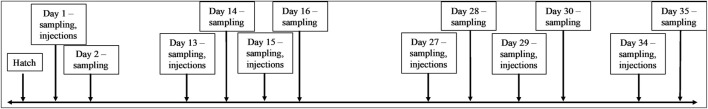
Timeline showing sampling and injection timepoints of trial.

Birds in two out of the total eight houses were assigned to one of four groups (modern broiler CpG treatment, modern broiler GpC control, ACRB CpG treatment or ACRB GpC control). GpC contains the same nucleotides as CpG but in an inverted, non-stimulatory, sequence (Ahmad-Nejad et al., 2002). The birds were given their first injection of either CpG or the control GpC as a 25-µg injection in 0.2 mL of 0.01 M sterile phosphate buffered saline (PBS, Sigma Aldrich, St. Louis, MO) 1-day post-hatch, this dose has been shown to be within the range necessary to elicit a measurable immune response (Ahmad-Nejad et al., 2002). Prior to injections, 5 birds from each group were sacrificed via cervical dislocation and their tissues were collected for analysis. Twenty-4 hours later, five additional birds from each group were sacrificed and their tissues were collected. This pattern of sampling, injecting, and sampling again was repeated on days 13, 14, 15, 16, 27, 28, 29, 30 and 34 and 35 post-hatch (Figure 1). The tissues collected at each sampling time point included jejunal samples and cecal tonsil samples. The tissue samples reserved for kinome analysis were flash frozen in liquid nitrogen and stored at −80°C until used for analysis. The tissue samples reserved for RNA extraction and qPCR were preserved in ∼1 mL of RNAlater, stored at 4°C overnight, then moved to −20°C until used for analysis.

### 2.3 Kinome array analysis

Jejunum and cecal tonsil tissue samples included in the analysis were from day 2, day 15, day 16, day 34, and day 35 post-hatch. The peptide array protocol using PepStar peptide microarrays from JPT Peptide Technologies GmbH (Berlin, Germany) was carried out as described previously (Arsenault, et al., 2017) and is summarized below with the listed modifications. Approximately 40 mg pieces of tissue were cut and homogenized by a Bead Ruptor homogenizer (Omni, Kennesaw GA) in 100 μL of lysis buffer (20 mM Tris–HCl pH 7.5, 150 mM NaCl, 1 mM Ethylenediaminetetraacetic acid (EDTA), 1 mM ethylene glycol tetraacetic acid (EGTA), 1% Triton X-100, 2.5 mM sodium pyrophosphate, 1 mM Na3VO4, 1 mM NaF, 1 μg/mL leupeptin, 1 g/mL aprotinin and 1 mM Phenylmethylsulphonyl fluoride). All chemicals were purchased from Sigma-Aldrich, Co. (St. Louis, MO) unless specified otherwise. Arrays were then imaged using a Tecan PowerScanner microarray scanner (Tecan Systems, San Jose, CA, USA) at 532–560 nm with a 580 nm filter to detect dye fluorescence. The kinome array images were gridded using the GenePix Pro seven software (Molecular Devices, LLC, San Jose, CA, USA), and the spot intensity signal was collected as the mean of pixel intensity using local feature background intensity calculation with the scanner saturation level set at 50%.

### 2.4 RNA extraction and qRT-PCR

RNA was isolated from the tissue samples (approximately 20 mg of tissue) using the Qiagen RNeasy MiniKit (Germantown, MD). The tissues were lysed in a 2 mL vial with 1.4 mm ceramic beads with 600 µL of buffer RLT using a Bead Ruptor 24 (Omni International, Kennesaw, GA) run on setting six for two cycles of 10 s. The isolated RNA was eluted into ultrapure water and stored at −80 °C until further analysis. RNA quality was verified by quantification on a NanoDrop 1,000 Spectrophotometer (Thermo Scientific, Waltham, MA) and gel electrophoresis of the RNA samples on an Invitrogen E-Gel EX 1% Agarose (Invitrogen, Carlsbad, CA). The RNA was used with the Applied Biosystems TaqMan RNA-to-CT 1-Step kit (Waltham, MA) following the protocol provided. The primers and probes for the housekeeping gene and cytokines of interest were ordered from Integrated DNA Technologies (Coralville, IA). PCR was performed on an Applied Biosystems 7900HT Real Time PCR System with a standard 96-well block. Each sample was run in triplicate.

### 2.5 Statistical analysis

Ross birds’ weights were measured as a group on days 3–12 post-hatch and then individually on days 15–34 post hatch. When the weights were taken individually a one-way Student’s t-test was used to compare the weights between the treatment and control birds and determine significant difference, and a *p*-value of <0.05 was considered significant. The ACRB birds were only weighed individually on days 29 and 34 post-hatch, at all other time points they were weighed as a group. When the ACRB birds were weighed individually, a one-way Student’s t-test was again used to compare weights between treatment and control birds.

The kinome data were analyzed using the PIIKA two peptide array analysis software (http://saphire.usask.ca/saphire/piika/index.html) (Trost et al., 2013). The resulting data points were normalized to eliminate variance due to technical variation such as random variation in staining intensity between arrays or between blocks within an array. Variance stabilization normalization was performed. There were three biological replicates for each sample, and the normalized fluorescence data for the three replicates were averaged together. PIIKA two then performed one-tailed T tests to determine the statistical significance of the change in phosphorylation at each peptide fragment between the treatment and the control tissues, generating a fold change and a *p*-value. Peptides with fold changes with a *p*-value <0.05 were considered statistically significantly differentially phosphorylated between treatment birds’ samples and control. In the rest of the manuscript the word “significant” when used in reference to kinome data will refer to these statistically significantly differentially phosphorylated peptides. Significant peptides/protein data was input into the protein-protein interaction database STRING (Szklarczyk et al., 2015) for interaction analysis and KEGG pathway analysis (Kanehisa et al., 2016). For the PCR data analyses the raw CT values from the qRT-PCR were used in a ΔΔCT calculation to determine fold change of mRNA expression between treatment and control tissue samples. A one-way ANOVA was conducted to determine if there was a statistical difference in fold-change of each cytokine in each tissue by day. If there was a significant different, a Student’s t-test with Bonferroni correction was performed to identify statistically significantly different fold changes.

## 3 Results

### 3.1 The modern broilers’ final weight is significantly impacted by the CpG treatment, heritage birds’ is not

**FIGURE 2:**
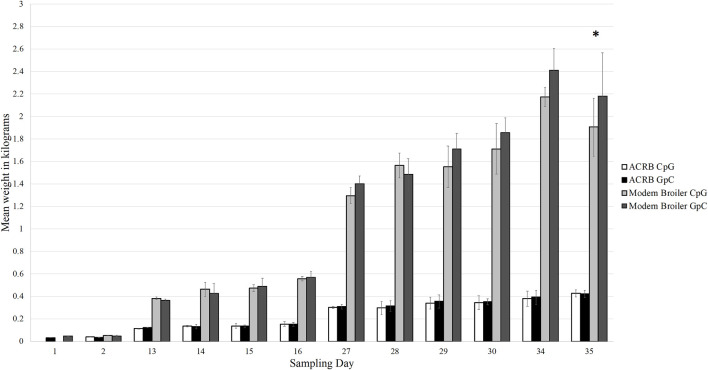
Bar graph showing the average weights of each bird type and treatment type at each sampling time point in the grow out period. The modern broilers, as expected, grew larger and faster than the ACRB birds. The difference in weights between the treatment and control birds was only significant in the modern broilers at day 35 post-hatch (denoted by the asterisk, *p* < 0.05). The difference in weight between the treatment and control ACRB birds was never significantly different from each other. The error bars indicate the standard deviation.

Through the grow-out period of 35 days, the modern birds grew more rapidly than the ACRB birds and had higher final body weights, as expected. On day 35 post-hatch, the control modern broilers had an average body weight of 2.18 kg, and the CpG injected modern broilers had an average body weight of 1.91 kg. This was the only sampling timepoint at which the difference in body weights between the treatment and control birds was statistically significant (Figure 2). The ACRB birds’ average body weight on day 35 post-hatch was 0.43 kg for both the control and treatment groups. At no point during the grow-out period was the difference in weights between control and treatment ACRB birds significant.

### 3.2 The significant peptides unique to the modern broiler’s day 2 cecal tonsil show innate signaling, heritage broiler day 2 cecal tonsil shows adaptive signaling

To characterize the different broilers’ response to the initial CpG injection, we compared the significant peptides in the CpG injected birds’ day 2 post-hatch cecal tonsils to the control birds’ in both the ACRB and the modern birds. We also repeated this comparison for the day 2 jejuna. When the day 2 ACRB and modern birds’ treatment and control samples were compared, differences in the two strains’ responses to the CpG treatment emerged. The list of significant peptides in the modern broiler day 2 cecal tonsils was compared to the list of significant peptides in the ACRB day 2 cecal tonsil, and this comparison was repeated for the jejuna samples. There were 58 significant peptides that were unique to the ACRB broilers’ jejuna samples on day 2 (after the first injection) post-hatch, compared to 199 significant peptides unique to the modern broilers’ jejuna samples (Supplementary Figure 1). When this analysis was performed using data from the cecal tonsil, there was not as great a disparity in the number of significant peptides unique to the ACRB birds’ day 2 samples and the modern broilers’ samples (107 and 99 respectively). To better understand the difference in each tissues’ responses to the CpG treatment, the significant peptides unique to the ACRB and modern broiler tissue samples were analyzed using STRING (Szklarczyk et al., 2015) to identify GO Biological processes (GO Bio) and KEGG pathways (Kanehisa et al., 2016) significantly enriched within the set of peptides (Tables 1, 2).

**TABLE 1:** The top 10 GO biological processes and KEGG pathways that were found via STRING analysis to be enriched within the set of significant peptides unique to the modern broiler day 2 cecal tonsils when compared to the list of significant peptides in the ACRB cecal tonsils.

Modern broiler day 2 cecal tonsil – GO terms				Modern broiler day 2 cecal tonsil – KEGG pathways			
pathway ID	pathway description	observed protein count	false discovery rate	pathway ID	pathway description	observed protein count	false discovery rate
GO.0016310	phosphorylation	30	3.81E-13	5200	Pathways in cancer	14	5.54E-08
GO.0007169	transmembrane receptor protein tyrosine kinase signaling pathway	25	5.95E-13	4152	AMPK signaling pathway	9	5.15E-07
GO.0006468	protein phosphorylation	26	8.64E-13	4010	MAPK signaling pathway	11	1.83E-06
GO.0006793	phosphorus metabolic process	35	9.72E-12	4015	Rap1 signaling pathway	10	2.20E-06
GO.0010646	regulation of cell communication	42	1.96E-11	4014	Ras signaling pathway	10	3.44E-06
GO.0023051	regulation of signaling	41	1.96E-11	4620	Toll-like receptor signaling pathway	7	1.59E-05
GO.0043085	positive regulation of catalytic activity	30	7.70E-11	4932	Non-alcoholic fatty liver disease (NAFLD)	8	1.59E-05
GO.0051128	regulation of cellular component organization	36	7.70E-11	4151	PI3K-Akt signaling pathway	10	0.000108
GO.0006796	phosphate-containing compound metabolic process	33	9.70E-11	5206	MicroRNAs in cancer	7	0.000129
GO.0051247	positive regulation of protein metabolic process	29	2.35E-10	30	Pentose phosphate pathway	4	0.000159

**TABLE 2:** The top 10 GO biological processes and KEGG pathways that were found via STRING analysis to be enriched within the set of significant peptides unique to the ACRB day 2 cecal tonsils when compared to the list of significant peptides in the modern broilers’ cecal tonsils.

ACRB day 2 cecal tonsil – GO terms				ACRB day 2 cecal tonsil – KEGG pathways			
pathway ID	pathway description	observed protein count	false discovery rate	pathway ID	pathway description	observed protein count	false discovery rate
GO.0010033	response to organic substance	37	1.14E-15	4660	T cell receptor signaling pathway	9	4.02E-09
GO.0071310	cellular response to organic substance	33	1.21E-15	4662	B cell receptor signaling pathway	8	4.02E-09
GO.0016310	phosphorylation	24	3.08E-12	4917	Prolactin signaling pathway	8	4.02E-09
GO.0070887	cellular response to chemical stimulus	32	3.08E-12	4014	Ras signaling pathway	11	5.34E-09
GO.0006468	protein phosphorylation	21	8.16E-12	4012	ErbB signaling pathway	8	1.10E-08
GO.0038095	Fc-epsilon receptor signaling pathway	13	8.16E-12	5205	Proteoglycans in cancer	10	6.74E-08
GO.0071495	cellular response to endogenous stimulus	23	8.16E-12	4722	Neurotrophin signaling pathway	8	9.52E-08
GO.0038093	Fc receptor signaling pathway	14	9.37E-12	4919	Thyroid hormone signaling pathway	8	9.52E-08
GO.0045087	innate immune response	22	1.02E-11	4010	MAPK signaling pathway	10	1.75E-07
GO.0071363	cellular response to growth factor stimulus	19	1.73E-11	5162	Measles	8	1.90E-07

In the list of pathways generated from significant peptides unique to the modern birds’ cecal tonsils, the MAPK, Toll-like receptor, and PI3k-Akt signaling pathways are among the top ten enriched pathways identified. In the ACRB birds’ cecal tonsils, however, the KEGG pathways enriched within the set of significant peptides unique to the ACRB day 2 cecal tonsil include more adaptive immune signaling pathways, such as the T and B cell receptor signaling pathways. In looking more closely at the proteins involved in the B and T cell receptor signaling pathways, there are indications of signaling activation or partial activation (Supplementary Table 1). The GO Bio terms enriched among the unique significant peptides unique to the ACRB day 2 cecal tonsil include the cellular response to organic substance (Table 2). Within the peptides enriched in this biological process, there are indications of cytoskeletal rearrangement, as well as lymphangiogenesis and in particular natural killer (NK) and innate lymphoid cell (ILC) development.

### 3.3 Significant peptides unique to the modern broiler day 2 jejunum show pro-survival signaling, heritage birds’ day 2 jejunum shows controlled innate immune response

In the ACRB birds’ day 2 jejunum, the phosphorylation patterns of the significant peptides involved in the innate immune response GO Bio process and the Toll-like receptor/PI3k-Akt/Insulin signaling pathways indicate negative feedback and control of an immune response, specifically negative feedback of the antiviral response (Tables 3, 4). In the modern broiler’s day 2 jejunum, conversely, much of the signaling activity corresponds to a pro-survival, non-apoptotic signaling, and immune signaling (Supplementary Table 2).

**TABLE 3:** The top 10 GO biological processes and KEGG pathways that were found via STRING analysis to be enriched within the set of significant peptides unique to the ACRB day 2 jejunum tissues’ when compared to the modern broilers’ significant peptides.

ACRB day 2 jejunum - GO Bio terms				ACRB day 2 jejunum - KEGG pathways			
pathway ID	pathway description	observed protein count	false discovery rate	pathway ID	pathway description	observed protein count	false discovery rate
GO.0006793	phosphorus metabolic process	19	0.00013	4620	Toll-like receptor signaling pathway	5	0.00181
GO.0016310	phosphorylation	15	0.00013	4910	Insulin signaling pathway	5	0.00333
GO.0006468	protein phosphorylation	13	0.000174	20	Citrate cycle (TCA cycle)	3	0.00492
GO.0006796	phosphate-containing compound metabolic process	18	0.000239	5164	Influenza A	5	0.00498
GO.0006955	immune response	15	0.000662	5020	Prion diseases	3	0.00633
GO.0009893	positive regulation of metabolic process	23	0.00173	1,200	Carbon metabolism	4	0.00676
GO.0010604	positive regulation of macromolecule metabolic process	20	0.00173	4014	Ras signaling pathway	5	0.0104
GO.0042325	regulation of phosphorylation	14	0.00173	4010	MAPK signaling pathway	5	0.0136
GO.0022407	regulation of cell-cell adhesion	8	0.00222	4621	NOD-like receptor signaling pathway	3	0.0136
GO.0045087	innate immune response	12	0.00222	5161	Hepatitis B	4	0.0136

**TABLE 4:** The top 10 GO biological processes and KEGG pathways that were found via STRING analysis to be enriched within the set of significant peptides unique to the modern broilers’ day 2 jejunum tissues’ when compared to the ACRB birds’ significant peptides.

Modern broiler day 2 jejunum – GO Bio terms				Modern broiler day 2 jejunum – KEGG pathways			
pathway ID	pathway description	observed protein count	false discovery rate	pathway ID	pathway description	observed protein count	false discovery rate
GO.0045087	innate immune response	55	4.34E-27	5200	Pathways in cancer	32	2.19E-21
GO.0050776	regulation of immune response	52	4.34E-27	4010	MAPK signaling pathway	25	9.20E-17
GO.0042325	regulation of phosphorylation	61	3.48E-26	4151	PI3K-Akt signaling pathway	28	9.20E-17
GO.0009893	positive regulation of metabolic process	95	8.32E-26	4722	Neurotrophin signaling pathway	18	1.82E-15
GO.0019220	regulation of phosphate metabolic process	65	8.32E-26	5205	Proteoglycans in cancer	21	6.52E-14
GO.0010604	positive regulation of macromolecule metabolic process	82	8.28E-25	5206	MicroRNAs in cancer	18	6.52E-14
GO.0031325	positive regulation of cellular metabolic process	84	1.91E-24	4015	Rap1 signaling pathway	20	1.89E-13
GO.0010033	response to organic substance	78	7.54E-24	4014	Ras signaling pathway	20	6.49E-13
GO.0001932	regulation of protein phosphorylation	56	8.96E-24	4620	Toll-like receptor signaling pathway	15	8.92E-13
GO.0002682	regulation of immune system process	59	1.79E-23	4910	Insulin signaling pathway	16	2.86E-12

### 3.4 At day 15/16 heritage broilers have an adaptive immune response to CpG injection in their cecal tonsils, metabolic signaling predominates in modern broilers’ cecal tonsils

Five birds from each group were sampled on day 15, then the remaining birds were given another injection of either CpG or GpC control as appropriate, and then five more birds from each group were sampled on day 16. The list of peptides that were significant prior to injection and the list of peptides significant after the injection for each tissue and bird type were compared to generate the Venn diagrams in Supplementary Figure 2. The CpG injection on day 15 led to a greater number of significant peptides in the modern broiler at day 16 in cecal tonsil than in the ACRB day 16 cecal tonsil (191 vs 83 respectively). The most striking impact of the CpG injection on day 15 is that in the ACRB birds’ day 16 cecal tonsil, the KEGG pathways enriched within the set of significant peptides unique to day 16 include adaptive and innate immune signaling pathways. In the modern birds’ day 16 cecal tonsil, conversely, the pathways enriched in the significant peptides unique to the post-injection time frame indicate a more metabolic response to the injection (Supplementary Table 3)

To better understand the immune response to the CpG injection in the ACRB birds’ cecal tonsils, the phosphorylation statuses of the peptides involved in all the top 10 KEGG signaling pathways enriched in the set of significant peptides unique to the day 16 cecal tonsil were examined. The phosphorylation patterns suggest potential T cell receptor signaling activation in the ACRB birds’ day 16 cecal tonsil (Table 5). In the modern birds’ day 16 cecal tonsil, however, the response to the CpG injection appears to be metabolic in nature. There is evidence of AMPK activation, increased insulin signaling, and mTOR inhibition in the modern birds’ day 16 cecal tonsil (Table 5).

**TABLE 5:** Top 10 enriched KEGG pathways in the set of significant peptides unique to day 16 (post-injection) in the modern broiler birds’ cecal tonsils (left) and in the significant peptides unique to day 16 (post-injection) in the ACRB birds’ cecal tonsils (right).

Top 10 KEGG pathways enriched in the significant peptides unique to the modern bird day 16 cecal tonsil				Top 10 KEGG pathways enriched in the significant peptides unique to the ACRB day 16 cecal tonsil			
Pathway ID	Pathway description	Observed gene count	False discovery rate	Pathway ID	Pathway description	Observed gene count	False discovery rate
hsa04910	Insulin signaling pathway	24	3.20E-20	hsa04380	Osteoclast differentiation	8	8.57E-06
hsa04010	MAPK signaling pathway	25	2.23E-14	hsa04510	Focal adhesion	9	1.00E-05
hsa05169	Epstein-Barr virus infection	20	6.81E-13	hsa04660	T cell receptor signaling pathway	7	1.10E-05
hsa05200	Pathways in cancer	29	1.04E-12	hsa05206	MicroRNAs in cancer	8	1.10E-05
hsa04931	Insulin resistance	16	1.20E-12	hsa04664	Fc epsilon RI signaling pathway	6	1.28E-05
hsa05161	Hepatitis B	17	4.14E-12	hsa04670	Leukocyte transendothelial migration	7	1.28E-05
hsa04152	AMPK signaling pathway	16	4.29E-12	hsa05145	Toxoplasmosis	7	1.28E-05
hsa04922	Glucagon signaling pathway	15	4.78E-12	hsa04152	AMPK signaling pathway	7	1.48E-05
hsa04380	Osteoclast differentiation	16	5.28E-12	hsa04650	Natural killer cell mediated cytotoxicity	7	1.62E-05
hsa04211	Longevity regulating pathway	14	1.23E-11	hsa04151	PI3K-Akt signaling pathway	10	2.23E-05

### 3.5 Modern broilers have enhanced immune signaling in the jejuna after treatment

In the jejuna samples, unlike the cecal tonsils, fewer significant peptides are unique to the day 16 post-injection time period than are common to pre- and post-injection or unique to pre-injection (Supplementary Figure 3). In the ACRB birds, there is a nearly equal number of significant peptides that are unique to the pre-injection time period and the post-injection time period. The top ten enriched KEGG pathways among the significant peptides unique to the modern birds’ post-injection jejuna samples show more indications of an adaptive immune response, however, the phosphorylation patterns show suppression of T and B cell receptor signaling activity (Table 6). In the ACRB birds, the significant peptides unique to the day 16 jejunum are more involved in metabolism and oxidative stress signaling, though there are indications of immune signaling as well (Table 6).

**TABLE 6:** Top 10 enriched KEGG pathways in the set of significant peptides unique to day 16 (post-injection) in the modern broiler birds’ jejunum (left) and in the significant peptides unique to day 16 (post-injection) in the ACRB birds’ jejunum (right).

Top 10 KEGG pathways enriched in the significant peptides unique to the modern bird day 16 jejunum				Top 10 KEGG pathways enriched in the significant peptides unique to the ACRB day 16 jejunum			
#term ID	term description	observed protein count	false discovery rate	#term ID	term description	observed protein count	false discovery rate
hsa04660	T cell receptor signaling pathway	10	3.07E-09	hsa00010	Glycolysis/Gluconeogenesis	8	6.71E-07
hsa04151	PI3K-Akt signaling pathway	14	1.31E-08	hsa04010	MAPK signaling pathway	13	6.71E-07
hsa04662	B cell receptor signaling pathway	8	5.45E-08	hsa04066	HIF-1 signaling pathway	9	6.71E-07
hsa04722	Neurotrophin signaling pathway	9	7.41E-08	hsa04662	B cell receptor signaling pathway	8	6.71E-07
hsa04152	AMPK signaling pathway	9	7.85E-08	hsa05200	Pathways in cancer	17	6.71E-07
hsa05200	Pathways in cancer	15	7.85E-08	hsa00052	Galactose metabolism	6	1.33E-06
hsa04910	Insulin signaling pathway	9	1.39E-07	hsa00500	Starch and sucrose metabolism	6	1.58E-06
hsa04510	Focal adhesion	10	1.97E-07	hsa04064	NF-kappa B signaling pathway	8	1.68E-06
hsa05161	Hepatitis B	9	1.97E-07	hsa05162	Measles	9	1.68E-06
hsa04620	Toll-like receptor signaling pathway	8	2.33E-07	hsa04620	Toll-like receptor signaling pathway	8	2.62E-06

### 3.6 The response to the injection is similar in both bird strains

By the time the birds reach the end of the sampling period, days 34 and 35 post-hatch, the response to the CpG injection decreases, as there are fewer significant peptides that are unique to the post-injection timepoint in both the modern birds’ and ACRB birds’ cecal tonsils (Supplementary Figure 4). There is still a larger proportion of the total significant peptides unique to the post-injection timepoint in the modern birds than in the ACRB birds.

In the ACRB birds’ cecal tonsils, there are only three to five proteins in the significantly enriched KEGG pathways generated from the list of unique significant peptides (Supplementary Table 4). Largely, the enriched pathways are involved in fatty acid oxidation, glucose metabolism, and T and B cell growth.

The significant peptides unique to the modern broiler’s day 35 (post-injection) cecal tonsil are enriched for KEGG pathways including autophagy, osteoclast differentiation, and viral response associated pathways. The phosphorylation patterns in these signaling pathways indicate response to unfolded protein stress. The B cell receptor pathway is enriched within the set of peptides unique to day 35, but the signaling patterns are suggestive of inhibition.

By day 35, the signaling changes in both the ACRB and modern birds’ jejuna are similar to each other. Among the top 10 enriched KEGG pathways in both tissues are the MAPK signaling pathway, the Insulin signaling pathway, Pathways in cancer, the Hepatitis B signaling pathway, and the PI3k-Akt signaling pathway (Tables 7, 8). There were also a similar number of significant peptides unique to the post-injection time point in the ACRB birds and the modern birds (105 and 117 respectively) (Supplementary Figure 5).

**TABLE 7:** Top 10 enriched KEGG pathways in the set of significant peptides unique to day 35 (post-injection) in the modern broiler birds’ cecal tonsils (left) and in the significant peptides unique to day 35 (post-injection) in the ACRB birds’ cecal tonsil (right).

Top 10 KEGG pathways enriched in the significant peptides unique to the modern bird day 35 cecal tonsil				Top 10 KEGG pathways enriched in the significant peptides unique to the ACRB bird day 35 cecal tonsil			
Pathway ID	Pathway description	Observed protein count	False discovery rate	Pathway ID	Pathway Description	Observed protein count	False discovery rate
hsa04140	Autophagy – animal	9	4.73E-07	hsa01200	Carbon metabolism	5	0.0022
hsa04380	Osteoclast differentiation	9	4.73E-07	hsa04910	Insulin signaling pathway	5	0.0022
hsa05160	Hepatitis C	9	4.73E-07	hsa05230	Central carbon metabolism in cancer	4	0.0022
hsa05167	Kaposi’s sarcoma-associated herpesvirus infection	10	4.73E-07	hsa04146	Peroxisome	4	0.0024
hsa05200	Pathways in cancer	14	5.99E-07	hsa00520	Amino sugar and nucleotide sugar metabolism	3	0.0086
hsa04662	B cell receptor signaling pathway	7	7.21E-07	hsa04068	FoxO signaling pathway	4	0.0094
hsa05162	Measles	8	2.17E-06	hsa04213	Longevity regulating pathway - multiple species	3	0.012
hsa05169	Epstein-Barr virus infection	9	2.35E-06	hsa05206	MicroRNAs in cancer	4	0.012
hsa05161	Hepatitis B	8	2.73E-06	hsa00010	Glycolysis/Gluconeogenesis	3	0.0127
hsa04066	HIF-1 signaling pathway	7	3.42E-06	hsa04920	Adipocytokine signaling pathway	3	0.0127

**TABLE 8:** Top 10 enriched KEGG pathways in the set of significant peptides unique to day 35 (post-injection) in the ACRB broiler birds’ jejunum (left) and in the significant peptides unique to day 35 (post-injection) in the modern broiler birds’ jejunum (right).

Top 10 KEGG pathways in the significant peptides unique to ACRB birds’ day 35 jejunum				Top 10 KEGG pathways in the significant peptides unique to modern broiler birds’ day 35 jejunum			
Pathway ID	Pathway description	Observed protein count	False discovery rate	Pathway ID	Pathway description	Observed protein count	False discovery rate
hsa04010	MAPK signaling pathway	16	5.94E-10	hsa04010	MAPK signaling pathway	18	1.87E-11
hsa04380	Osteoclast differentiation	10	1.38E-07	hsa04910	Insulin signaling pathway	14	1.87E-11
hsa05230	Central carbon metabolism in cancer	8	2.08E-07	hsa05167	Kaposi’s sarcoma-associated herpesvirus infection	15	2.08E-11
hsa05161	Hepatitis B	10	2.36E-07	hsa05161	Hepatitis B	13	1.77E-10
hsa04931	Insulin resistance	9	2.73E-07	hsa05200	Pathways in cancer	20	5.91E-10
hsa05200	Pathways in cancer	16	2.79E-07	hsa05215	Prostate cancer	11	6.95E-10
hsa04014	Ras signaling pathway	11	8.69E-07	hsa05169	Epstein-Barr virus infection	13	3.90E-09
hsa04151	PI3K-Akt signaling pathway	13	8.69E-07	hsa04151	PI3K-Akt signaling pathway	16	4.64E-09
hsa04910	Insulin signaling pathway	9	9.50E-07	hsa04657	IL-17 signaling pathway	10	5.76E-09
hsa04620	Toll-like receptor signaling pathway	8	1.64E-06	hsa05160	Hepatitis C	11	8.24E-09

### 3.7 The cytokine expression data indicate initial robust response that tapers off

**FIGURE 3:**
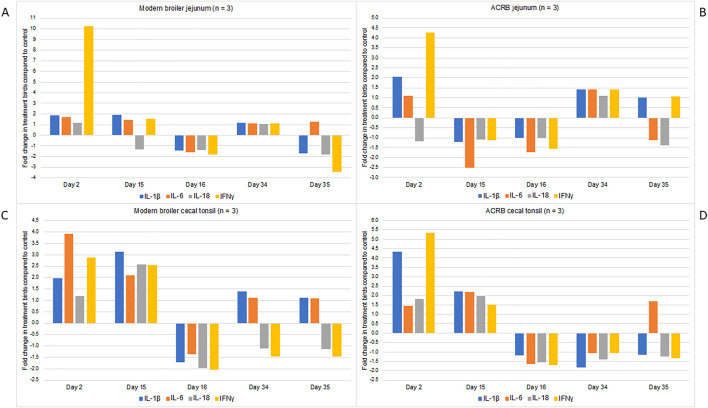
RNA was isolated from three individual birds’ cecal tonsil and jejunum tissue samples and used for RT-PCR. Analyses were performed using primers for IL-1beta, IL-6, IL-18 and IFN-gamma. The data are presented here as fold-changes between the treatment birds and the control, i.e., numbers greater than one indicate a fold-increase, and negative numbers indicate a fold-decrease in treatment compared to control birds. Fold changes values between 0 and one were converted into the reciprocal negative number. **(A)** Shows gene expression data from the modern broiler jejunum tissue, **(B)** ACRB jejunum tissue, **(C)** modern broiler cecal tissue, and **(D)** ACRB cecal tissue.

In both tissues and both bird types, the expression of proinflammatory cytokines is increased immediately following the first injection with CpG (Figure 3). There are differences in which cytokines are increased in each tissue, and the degree to which the expression increases. It is interesting that the increased cytokine expression continues to the day 15 post-hatch time point in the modern birds’ cecal tonsil, but then following the injection on day 15 this expression drops off below the control birds. For all other tissues and time points in both birds the expression of each cytokine is similar to control or below control birds’ levels. A one-way ANOVA was conducted to identify significant changes in fold change expression by day. In the modern broiler jejunum, the fold change in IFN-γ expression was significantly different on day 2 post-hatch compared to all other sampling time points. Similarly, in the modern birds’ cecal tonsil the fold change in IL-1β expression was significantly different on day 2 post-hatch when compared with other sampling days. In the ACRB cecal tonsil, the fold change in IL-1β on day 2 post hatch was significantly different from that of other sampling days (day 15 post-hatch excepted). In the ACRB jejunum, the fold change in expression of IFN-γ was significantly different on day 2 post hatch from any other sampling day.

## 4 Discussion

The cecal tonsils are the largest organ in the avian gut-associated lymphoid tissue (GALT), and home to many lymphocyte populations from which cell-mediated and humoral immune responses could influence the immune system in the rest of the gastrointestinal tract. The signaling patterns in the modern broilers cecal tonsils on the day after the first injection were indicative of innate immune signaling as shown by the significantly enriched TLR signaling In the modern birds’ day 2 cecal tonsil, there are indications of active TLR signaling; signaling proteins such as specific MAPKs, RAC1, and the phosphatase DUSP6 all show phosphorylation patterns that would result downstream of TLR ligation. When mononuclear cells are isolated from commercial broiler chicks’ cecal tonsils and treated *in vitro* with TLR ligands, including LPS isolated from *E. coli* (*Escherichia coli*) O 111:B4 and CpG, the stimulation of the TLRs leads to increased mRNA expression of proinflammatory cytokines such as IFN-γ, IL-1β and IL-6 (Taha-abdelaziz et al., 2016). The broilers treated with CpG in this study showed similar increases in cytokine mRNA in their cecal tonsils after the first exposure. There are also indications of non-TLR specific innate signaling, such as activated receptors that would lead downstream to increased hematopoiesis of innate immune cells such as monocytes and dendritic cells. This signaling coupled with the increased proinflammatory cytokines suggest a primarily innate response occurred in the modern birds’ cecal tonsils the day after the first treatment with CpG.

In the ACRB birds, conversely, two of the top 10 KEGG pathways significantly enriched among the peptides unique to the ACRB birds are the B cell receptor signaling pathway and the T cell receptor signaling pathway, the main cell types that constitute an adaptive immune response. The significantly differentially phosphorylated proteins are highly specific for either the B or T cell receptor signaling pathways. Zap70, for one, is a src-family kinase that is highly characteristic of T cell receptor signaling (Fischer et al., 2010), and it is uniquely significant in the ACRB birds’ cecal tonsil. Bruton’s tyrosine kinase (BTK) is another significant protein unique to the ACRB birds’ cecal tonsil that is characteristic of B cell receptor signaling (Kurosaki and Kurosaki, 1997). Our data suggest that there are populations of B and T cells in the ACRB broilers’ day 2 post-hatch cecal tonsil that potentially respond to the TLR21 stimulation.

There are also increases in proinflammatory cytokine mRNA in the ACRB birds’ day 2 cecal tonsil, although only IL-1β and IFN-γ showed significant fold increases. IFN-γ is a proinflammatory cytokine that participates in the Th1 effector cell differentiation process and immune response (Morales-Mantilla and King, 2018). The increase in IFN-γ production along with T cell signaling suggest there is the possibility for this type of response in the ACRB birds’ cecal tonsils. Pro-IL-1β needs to undergo cleavage by caspase 1 (Casp1) to become the final IL-1β protein (Denes et al., 2012). Casp1 is significantly dephosphorylated in the ACRB day 2 cecal tonsil, though this is at a site with no downstream implications of the phosphorylation yet elucidated, so the impact of this Casp1 phosphorylation is unknown.

In the modern broilers’ jejunum on day 2 post-hatch, after the first injection of CpG, the signaling patterns indicate anti-apoptotic signaling as well as cell-cycle progression. Specifically, Caspase-3 is inhibited in the modern birds’ jejunum. This caspase has been linked to neutrophil apoptosis during the contraction of an inflammatory response (Alvarado-Kristensson et al., 2004). Avian species have heterophils rather than neutrophils, but these cells have a similar function and phenotype to mammalian neutrophils. Potentially, their apoptosis processes could be controlled by a similar mechanism (Horn et al., 2012). This could be indicative of potentially increased heterophil longevity within the modern birds’ jejunum as a response to the CpG. Modern broilers demonstrate robust cell-mediated immune responses (Cheema et al., 2003), and the inhibition of apoptosis and increased cell-cycle signaling observed may be indicative of that process.

In the ACRB birds, the significant peptides unique to the day 2 jejunum are suggestive of negative feedback and feedback inhibition of immune responses. TBK1 is phosphorylated at a site that the kinase Lck would target for phosphorylation that would lead to inhibition of TBK1 as part of the negative feedback response (Liu et al., 2017). Lck is also phosphorylated on an activation site in these tissues, so negative feedback signaling as part of the antiviral response may be ongoing. Furthermore, NFIL3 is phosphorylated on a negative regulatory site (Table 6), and this protein is important for the development of ILCs and NK cells (Kostrzewski et al., 2018). It is interesting that in both the modern and ACRB broiler’s day 2 jejunum the biggest change in cytokine expression after the first injection is a fold-increase in IFN- γ expression. CpG treatment is known to induce IFN-γ production in avian PBMCs, so this raises the question of which resident cells in the avian jejunum are producing this cytokine in response to the CpG treatment (He et al., 2003).

At the day 15/16 post-hatch experimental time point, the pattern seen earlier in the cecal tonsils is recapitulated, where the response in the ACRB birds mostly resembles adaptive immune signaling, however the response in the modern birds involves more metabolic signaling. Additionally, there is a larger number of significant peptides unique to the day 16 timepoint in the modern birds’ cecal tonsil than the heritage bird. These data suggest that the impact of the CpG injection on day 15 was greater in the modern birds than in the heritage birds. In the ACRB birds’ tissues at the post-injection time point there is partial activation along the T/B cell receptor signaling pathway and there is also activation of focal adhesion signaling pathways and cytoskeletal rearrangement. Focal adhesion and cytoskeleton rearrangement are important for T cell activation and signaling (Rozdzial et al., 1995) and activation of these pathways may further suggest T cell activation after the day 15 CpG injection. The metabolic state of immune cells has also been shown to directly influence their activation and differentiation (Shi et al., 2011; Macintyre et al., 2014). In the case of the ACRB birds’ day 16 cecal tonsil, mTOR is significantly dephosphorylated on its activation sites, but the ribosomal protein S6 kinase beta-1 is significantly phosphorylated on an activation site (Table 8), where it would be phosphorylated by active mTOR (Saitoh et al., 2002). However, this phosphorylation can be accomplished by other kinases, such as PDK1, so it is still possible that mTOR is inactivated in these tissues. There is also evidence of TGF-β signaling ongoing in these tissues. The combination of TGF-β signaling, T cell receptor activation, and mTOR inhibition could be indicative of the generation of regulatory T cells (Ghiringhelli et al., 2005; Delgoffe et al., 2009). Tregs are important modulators of inflammatory responses, and especially so in the intestinal environment where there are large populations of commensal bacteria and unwarranted inflammation could be damaging to the surrounding tissues. These data may indicate ACRB broilers’ generating regulatory T cells to temper inflammation in response to the CpG treatment given on day 15 post-hatch. The PCR data support the potential for a regulatory environment, as there are no increases in expression of the pro-inflammatory cytokines in response to the day 15 injection.

In the modern birds’ cecal tonsils after the day 15 injection metabolic signaling pathways are enriched in the significant peptides unique to the post-injection timepoint. Subunits of AMPK are significantly phosphorylated on target sites that play a role in AMPK localization and confer the potential for activation. Acetyl-CoA carboxylase is also significantly phosphorylated, which would be activated downstream of AMPK activity (Table 8). mTOR is also inhibited in these tissues, but there is activation of insulin signaling. These metabolic shifts indicate that there could be a need in these cells for increased cellular energy. AMPK is a master energy sensor in cells and is activated when cells sense a high AMP: low ATP ratio (Long and Zierath, 2006). Part of AMPK’s response to low cellular energy is to inhibit processes that consume energy, such as protein synthesis, so AMPK inhibits mTOR activation. Raptor is a regulatory protein that controls mTOR activation and in these tissues it is significantly dephosphorylated at a site that would be phosphorylated by JNK1 in response to oxidative stress (Table 8) (Kwak et al., 2012). This JNK1 mediated phosphorylation of Raptor in response to stress would lead to mTOR activation, and the significant dephosphorylation of Raptor in the modern birds’ day 16 cecal tonsils could indicate further inhibition of mTOR. The cytokine mRNA expression on day 16 supports the kinome data as the expression drops off considerably compared to the data from day 15 and drops below control.

In the modern birds’ jejuna there is immune signaling enriched among the significant peptides unique to this time point (Table 8). This signaling is inhibitory and the cytokine data reflect that pattern as well (Figure 3). While the CpG has a more dynamic impact on signaling in the modern birds’ cecal tonsil than the heritage birds’, that is not the case in the jejunum.

By the end of the experimental period, the ACRB and modern broiler’s responses to the injection are similar, with many of the same pathways enriched specifically in the post-injection timepoint in the jejunum samples. However, in the ACRB birds’ cecal tonsils, uniquely, there is signaling that indicates increased fatty acid oxidation. Innate immune cells can undergo epigenetic changes in response to repeated exposure to non-specific pathogenic stimuli, and these changes can be linked to alterations in metabolism (Bekkering et al., 2018).

In summary, the modern broilers’ jejunum, responded to the treatment with changes to metabolic signaling pathways (Table 5). In the ACRB, adaptive immune signaling was enriched specifically in their cecal tonsils (Table 4). ACRB jejuna also showed immune signaling changes in response to the treatment, although there were additional changes in carbohydrate metabolism and indications of oxidative and metabolic stress. The modern broilers’ cecal tonsils showed a consistently greater magnitude of response to the CpG treatment, with more significant peptides unique to the post-injection timepoint in the modern birds than the ACRB birds. After the first injection, both modern and ACRB broilers had increased mRNA expression of various pro-inflammatory cytokines in both their jejuna and cecal tonsils in the treatment birds compared to the control birds (Figure 3). After the subsequent injections, however, the cytokine mRNA expression did not exhibit large changes in expression between the treatment and control birds; indeed, the mRNA expression of the cytokines measured often decreased in the treatment birds compared to that of the control birds’ post-injection.

## 5 Conclusion

Overall, the ACRB birds’ responses to the repeated injections with CpG indicate initiation of adaptive immune responses early on, that then mature into regulatory responses. The modern birds’ immune signaling indicates a more innate response initially, coupled with a metabolic shift in the cecal tonsil, the major lymphoid organ of the chicken GALT. Strong evidence for this is the reduced growth observed in the modern broilers while the ACRBs showed no detrimental effect on their growth from the repeated CpG injections.

The data presented in this study showcase the need to better understand the allocation of resources, and specifically energy in the modern broiler, a bird that is genetically programmed for rapid growth and yet needs the metabolic flexibility to support growth and effective immune functions. This balance is especially important during the time in broiler chickens’ life when they are both experiencing accelerating growth and more susceptible to disease challenges, which coincide at approximately the 2-week post-hatch mark. Better understanding of this vulnerable time can potentially lead to therapeutic interventions to preserve bird health and efficiency in a post-antimicrobial industry.

## Data Availability

The original contributions presented in the study are included in the article/Supplementary Material, further inquiries can be directed to the corresponding author.
