# Supplementary material for: Modern broiler chickens exhibit a differential gastrointestinal immune and metabolic response to repeated CpG injection relative to a 1950s heritage broiler breed
Source: Front Physiol. 2024 Nov 1;15:1473202. doi: 10.3389/fphys.2024.1473202 (PMC11565619; doi:10.3389/fphys.2024.1473202)
Supplement: Supplementary file 1 [file Table4.pdf]

Supplementary Table 4: List of significant peptides and summary of their activation statuses unique to the day 35 ACRB cecal tonsil when compared to the significant peptides in the ACRB day 34 cecal tonsil. The same comparison is repeated for the modern broiler day 34 and day 35 cecal tonsil. The arrows indicate the direction of phosphorylation (up arrow, phosphorylation, down arrow, dephosphorylation) at a given phosphorylation target site on a peptide fragment corresponding to the protein indicated.

**Proteins uniquely significant**	**ACRB day 35 cecal tonsil phosphorylation at each site**	**Activation state**	**Proteins uniquely significant**	**Modern broiler day 35 cecal tonsil phosphorylation at each site**	**Activation state**
ALDOB	↓	No activity affiliated	BLNK	↓↓	Inactive (Fu et al., 1998)
CAT	↓ -	Inactive (Cao et al., 2003)	CAMKK2	↑	Phosphorylation here inhibits CAMKK autophosphorylation (Schumacher et al., 2004)
CBL	- ↓	Inactive (Thien et al., 2010)	CASP8	↑	Inhibited (Alvarado-Kristensson et al., 2004a)
CROT	↓	No activity affiliated	CDK6	↓ -	Not inhibited (Bertero et al., 2013, p. 25)
CS	↑	No activity affiliated	EIF2AK2	↓↑	Partial enzymatic activation (Taylor et al., 2001; Dar et al., 2005)
FGFR1	↑↑	Activated, (Furdui et al., 2006, p. 1)	EIF2AK3	- ↑ -	Activation, inhibition of protein synthesis (Kebache et al., 2004, p. 1)
HDAC4	↑↓	Prevented from leaving nucleus (Zhang et al., 2017), not held in cytoplasm (Wang et al., 2000)	EIF2AK4	- ↑	No activity affiliated with significant phosphosite
HKDC1	↓	No activity affiliated	FOS	↓	Destabilized by lack of phosphorylation here (Bakiri et al., 2011)
HSD17B4	- ↑↓	No activity affiliated	HKDC1	↑	No activity affiliated
IGF2BP1	↑	No activity affiliated	HRAS	↓	Inactive, apoptosis not induced (Kinoshita et al., 1997)
MARCKS	↓	Not active, potentially has a role in immune cell migration/infiltration (Chen et al., 2015)	HSP90B1	↓	No activity affiliated
NTRK1	- - ↑	Activation (Stephens et al., 1994)	IKBKE	↓	Inactive (Zhang et al., 2016)
PDK1	↓ -	Not active (Caohuy et al., 2014)	IL23R	↑	No activity affiliated
PECR	↑	No activity affiliated	MLST8	↑	No activity affiliated
PGM1	↑	Active (Gururaj et al., 2004)	NCF2	↑	No activity affiliated
PGM3	↓	No activity affiliated	NFKB2	↓	Not targeted for processing (Xiao et al., 2001)

PRKAA2	↑	No activity affiliated	NR1H3	↑	No activity affiliated with significant phosphosite
PRKAB2	↑ -	No activity affiliated, may be similar to PRKAB1 (Chen et al., 1999)	PDK1	- ↓	Not active (Caohuy et al., 2014)
PRKAR1B	↓	No activity affiliated	PFKFB3	↑	Active, can lead to increased glycolysis (Bando, 2005)
STAT3	↑ -	Activation (Song et al., 2018)	PIK3AP1	↑	No activity affiliated
SUCLG2	- ↓ -	No activity affiliated	PIK3CD	↓	Not inhibited, can perform lipid kinase activities (Vanhaesebroeck et al., 1999)
			PLCG2	↑	Phosphorylated downstream of BCR engagement (Kim et al., 2004)
			PPARA	↓ -	Inactive (Wood et al., 2011)
			PPARG	↑	Targeted for degradation (Floyd and Stephens, 2002)
			PRKCQ	↑	Active, able to translocate & participate in TCR signaling (Thuille et al., 2005)
			RAB7A	↑	unable to traffic to the membrane (Shinde and Maddika, 2016)
			RHOA	↓	Not inhibited (Ellerbroek et al., 2003)
			RPS6KB1	↑ - -	Partial activation (Feng et al., 2008)
			SYK	↑ ↓ ↓	Adhesion activated, signaling deactivated(Tsang et al., 2008; Chang et al., 2012)
			TBK1	↓	Inactive (Liu et al., 2017)
			TGFBR1	↑	Active (Wieser et al., 1995)
