# Supplementary material for: Modern broiler chickens exhibit a differential gastrointestinal immune and metabolic response to repeated CpG injection relative to a 1950s heritage broiler breed
Source: Front Physiol. 2024 Nov 1;15:1473202. doi: 10.3389/fphys.2024.1473202 (PMC11565619; doi:10.3389/fphys.2024.1473202)
Supplement: Supplementary file 2 [file Table1.pdf]

Supplementary Table 1: List of significant peptides and summary of their activation statuses unique to the day 2 ACRB cecal tonsil when compared to the significant peptides in the modern broiler day 2 cecal tonsil and vice versa. The arrows indicate significantly increased (up arrow) or significantly decreased (down arrow) phosphorylation at a given phosphorylation target site on a peptide fragment corresponding to the protein indicated.

**Proteins uniquely significant**	**ACRB day 2 cecal tonsil phosphorylation at each site**	**Activation status**	**Proteins uniquely significant**	**Modern bird day 2 cecal tonsil phosphorylation at each site**	**Activation status**
BTK	- ↑	Active (Mahajan et al., 2001)	CASP8	↓	Not inhibited (Alvarado-Kristensson et al., 2004a)
FOS	↓	Destabilized by lack of phosphorylation here (Bakiri et al., 2011)	MAP2K3	↓	Inactive (Raingeaud et al., 1996, p. 3)
HRAS	↑	Pro-apoptotic (Kinoshita et al., 1997)	MAP2K4	↓↓	Not inhibited, but not activated (Schuringa et al., 2000; Park et al., 2002)
MAP2K2	↓↓	Inactive, can't interact with Akt (Procaccia et al., 2017)	MAPK8	↓	Not active (Dérijard et al., 1994, p. 1)
MAP3K7	↓ -	Inactive, possibly part of negative feedback control (Singhirunnusorn et al., 2005; Yang et al., 2014)	RAC1	↓	Not inhibited (Kwon et al., 2000)
NFKB1	- ↓	Stable, not targeted for proteolysis (Lang et al., 2003)	TLR1	↓	No activity affiliated
PAK2	↓	Phosphorylated transiently in stimulated neutrophils, block interaction with guanine nucleotide exchange factor (Zhan et al., 2003)	TLR5	↑	Active, response to flagellin (Ivison et al., 2007)
PIK3AP1	↓	No activity affiliated	DUSP6	↑	Destabilized, allows for ERK signaling (Jurek et al., 2009)
PIK3CD	↓	Not inhibited, can perform lipid kinase activities (Vanhaesebroeck et al., 1999)	CSF1R	↑ -	Activity induced (Rohde et al., 2004)

PLCG2	↑	Phosphorylated downstream of BCR engagement (Kim et al., 2004)	PPP2CA	↓	Not inhibited, could be active (Yokoyama et al., 2001)
PRKCQ	↑	Active, important in T lymphocyte function (Thuille et al., 2005)	RASSF5	↓	No activity affiliated; protein involved in lymphocyte adhesion
ZAP70	↑↓	Partial activation (Gong et al., 2001)	PFKL	- ↑	Active (Yugi et al., 2014)
FGFR1	↓↓	Not active (Furdui et al., 2006)	SIRT1	- ↑ -	Partial activation of enzymatic function (Nasrin et al., 2009, p. 1)
FLT4	↑	Active, required for kinase activity (Salameh et al., 2005)	SREBF1	↑	Negative regulation by PKA (Lu and Shyy, 2006)
RAB5A	↓	Not active, not involved in T lymphocyte migration, would be phosphorylated by ERK (Ong et al., 2014, p. 5)	MAP3K5	↑	Active, part of innate immune signaling (Cho et al., 2015)
SHC3	↓	No activity affiliated	RAF1	↓↓↓	Dephosphorylated by active PPP2CA, allows for active signaling (Dougherty et al., 2005)
