# Supplementary material for: Modern broiler chickens exhibit a differential gastrointestinal immune and metabolic response to repeated CpG injection relative to a 1950s heritage broiler breed
Source: Front Physiol. 2024 Nov 1;15:1473202. doi: 10.3389/fphys.2024.1473202 (PMC11565619; doi:10.3389/fphys.2024.1473202)
Supplement: Supplementary file 3 [file Table2.pdf]

Supplementary Table 2: List of significant peptides and summary of their activation statuses unique to the day 2 ACRB jejunum when compared to the significant peptides in the modern broiler day 2 jejunum and vice versa. The arrows indicate significantly increased (up arrow) or significantly decreased (down arrow) phosphorylation at a given phosphorylation target site on a peptide fragment corresponding to the protein indicated.

| **Proteins uniquely significant** | **ACRB day 2 jejunum phosphorylation at each site** | **Activation status** | **Proteins uniquely significant** | **Modern broiler day 2 jejunum phosphorylation at each site** | **Activation status** |
|---|---|---|---|---|---|
| TBK1 | ↓ | Not active (Li et al., 2017) | BRAF | ↓ - ↓ | Inhibited (MacNicol et al., 2000) |
| NFIL3 | ↑ | Inhibited (Kostrzewski et al., 2018) | CASP3 | ↑ | Inhibited (Alvarado-Kristensson et al., 2004a) |
| ETS1 | ↓ | Not active (Yang et al., 1996) | CCNE1 | ↓ | Not destabilized , cell cycle proceeding (Won and Reed, 1996) |
| NLRP3 | ↑ | No activity affiliated | CDK6 | ↓↓ | Not inhibited (Bertero et al., 2013, p. 25) |
| TOLLIP | ↑ | No activity affiliated | CRK | ↓↓↓ | Inactive (Sriram et al., 2015) |
| | | | GRB2 | ↓ | Not inhibited (Li et al., 2001) |
| | | | GSK3B | ↑↑↓ | Activation (Song et al., 2002; Zhang et al., 2011) |
| | | | HSP90AB1 | ↑↑ | Active, can lead to apoptosis (Kurokawa et al., 2008) |
| | | | IFNAR1 | ↑ | Active (Kumar et al., 2004) |
| | | | IKBKE | ↑ | Activated, plays a role in controlling T cell responses (Zhang et al., 2016) |
| | | | IL7R | ↑ | Active, (Venkitaraman and Cowling, 1994, p. 7) |
| | | | MAP2K4 | - ↓ | Inactive (Schuringa et al., 2000, p. 6) |
| | | | MET | ↑↑↓ | Partial activation (Bardelli et al., 1999) |
| | | | PIK3AP1 | ↓ | No activity affiliated |
| | | | PIK3CD | ↑ | Lipid kinase activity inhibited (Vanhaesebroeck et al., 1999) |
| | | | PLCG2 | ↑ | Phosphorylated downstream of BCR engagement (Kim et al., 2004) |

| | | | | | |
|---|---|---|---|---|---|
| | | | PTEN | ↑ - - | Active (Koul et al., 2002) |
| | | | TAB1 | ↓ | (Singhirunnusorn et al., 2005) |
| | | | YWHAZ | ↓ | Inhibited (Zhou et al., 2009) |
