# Supplementary material for: Modern broiler chickens exhibit a differential gastrointestinal immune and metabolic response to repeated CpG injection relative to a 1950s heritage broiler breed
Source: Front Physiol. 2024 Nov 1;15:1473202. doi: 10.3389/fphys.2024.1473202 (PMC11565619; doi:10.3389/fphys.2024.1473202)
Supplement: Supplementary file 4 [file Table3.pdf]

Supplementary Table 3: List of significant peptides and summary of their activation statuses unique to the day 16 ACRB cecal tonsil when compared to the significant peptides in the ACRB day 15 cecal tonsil. The same comparison is repeated for the modern broiler day 15 and day 16 cecal tonsil. The arrows indicate the direction of phosphorylation (up arrow, phosphorylation, down arrow, dephosphorylation) at a given phosphorylation target site on a peptide fragment corresponding to the protein indicated.

**Proteins uniquely significant**	**ACRB day 16 cecal tonsil phosphorylation at each site**	**Activation status**	**Proteins uniquely significant**	**Modern broiler day 16 cecal tonsil phosphorylation at each site**	**Activation status**
FYN	↑↑	Partial activation (Mustelin et al., 1992; Filipp et al., 2003)	ACACA	↑ - ↑	Inhibited (Hunkeler et al., 2018)
GRB2	↓	Not inhibited (Li et al., 2001)	ACACB	↑↑	No activity affiliated
JAK1	- ↑	Active (Wang et al., 2003)	AKT1	↑↓	Partial activation (Yang et al., 2015)
JAK2	- - ↑	Active (Sathyanarayana et al., 2012, p. 1)	AKT3	↑↑	Phosphorylated downstream of insulin signaling (Nakatani et al., 1999)
MAPK14	↑ -	Partial activation (Zhang et al., 2011)	BRAF	↓↓↑	Partial activation (MacNicol et al., 2000)
PAK2	↑	Phosphorylated transiently in stimulated neutrophils, block interaction with guanine nucleotide exchange factor (Zhan et al., 2003)	EIF4EBP1	- ↓ -	Not inhibited, repressing translation (Lekmine et al., 2004, p.)
PLCG2	↑	Phosphorylated downstream of BCR engagement (Kim et al., 2004)	GRB2	↓	Not inhibited (Li et al., 2001)
PPP2CA	↓	Not inhibited, could be active (Yokoyama et al., 2001)	GSK3B	- - ↓	Not inhibited (Song et al., 2002; Zhang et al., 2011)
PTK2	↑ - ↑	Active (Chen et al., 1996)	HK1	↓ - -	No activity affiliated
SYK	↑ - ↓	Adhesion activation, not signaling (Tsang et al., 2008;	HK2	↑↑ -	No activity affiliated

		Chang et al., 2012)			
NOS2	↑	Inhibited (Hausel et al., 2006)	HKDC1	↑	No activity affiliated
TGFBR1	↑	Active (Wieser et al., 1995)	INSR	↑	Active (Roskoski, 2017)
VCL	↑	No activity affiliated	MAPK8	↑	Active (Dérijard et al., 1994, p. 1)
EZR	↓↓	Inactive (Babich and Di Sole, 2015)	MTOR	↓ - ↓	Inactive, (Soliman et al., 2010)
JAM3	↑	No activity affiliated	PIK3CD	↑	Lipid kinase inhibited, phosphorylated downstream of TCR signaling (Vanhaesebroeck et al., 1999)
ALOX5	↑ -	Active (Hanaka et al., 2005)	PIK3CG	↑↑↑	Inhibited (Perino et al., 2011)
CASP8	↓	Not inhibited (Alvarado-Kristensson et al., 2004a)	PKLR	- ↓	No activity affiliated
CD44	↓↓	Cell migration deactivated (Peck and Isacke, 1998)	PPARGC1A	↓ - ↓	Destabilization, deactivation (Puigserver et al., 2001)
CAPN2	↓	Not inhibited (Shiraha et al., 2002)	PRKAA1	↑ - -	No activity affiliated with significant phosphosite, activation sites are more phosphorylated in treatment than in control, but not significantly (P = 0.25) (Puustinen et al., 2020)
ACACB	↓ -	No activity affiliated	PRKAB1	- ↑	Anchors protein in extranuclear space, potentially anchors AMPK alpha subunit as well (Warden et al., 2001)
CAMKK2	↑	Phosphorylated by death-associated protein kinase (Schumacher et al., 2004)	PRKAB2	- ↓	No activity affiliated, may be similar to PRKAB1 (Chen et al., 1999)
CPT1A	↑	No activity affiliated	PRKACA	↑↑	Active (Langer et al., 2005)
NFATC2	↑	No activity affiliated	PTPN1	↑	Active, phosphorylation allows for interaction with insulin receptor

					(Bandyopadhyay et al., 1997)
			PYGL	- ↑ -	No activity affiliated with significant phosphosite
			RAF1	↓ - -	Dephosphorylated by active PPP2CA, allows for active signaling (Dougherty et al., 2005)
			RPTOR	↓ - -	Inactive, not contributing to mTOR activation (Frey et al., 2014)
			RPS6KB1	↓ - ↓	Not active (Feng et al., 2008)
			SOCS3	↑ -	Active (Qasimi et al., 2006)
			SREBF1	↑	Negative regulation by PKA (Lu and Shyy, 2006)
			TSC2	↑ - ↑	Inhibited, would not inhibit mTOR signaling (Inoki et al., 2002)
