# Supplementary material for: Modern broiler chickens exhibit a differential gastrointestinal immune and metabolic response to repeated CpG injection relative to a 1950s heritage broiler breed
Source: Front Physiol. 2024 Nov 1;15:1473202. doi: 10.3389/fphys.2024.1473202 (PMC11565619; doi:10.3389/fphys.2024.1473202)
Supplement: Supplementary file 5 [file DataSheet1.pdf]

Supplementary References

Babich, V., and F. Di Sole. 2015. The Na+/H+ Exchanger-3 (NHE3) Activity Requires Ezrin Binding to Phosphoinositide and Its Phosphorylation. PLoS ONE 10 Available at https://www.ncbi.nlm.nih.gov/pmc/articles/PMC4455992/ (verified 17 April 2020).

Bakiri, L., M. O. Reschke, H. A. Gefroh, M. H. Idarraga, K. Polzer, R. Zenz, G. Schett, and E. F. Wagner. 2011. Functions of Fos phosphorylation in bone homeostasis, cytokine response and tumorigenesis. Oncogene 30 Available at https://www.ncbi.nlm.nih.gov/pmc/articles/PMC3838948/ (verified 16 April 2020).

Bando, H. 2005. Phosphorylation of the 6-Phosphofructo-2-Kinase/Fructose 2,6-Bisphosphatase/PFKFB3 Family of Glycolytic Regulators in Human Cancer. Clin. Cancer Res. 11:5784–5792.

Bandyopadhyay, D., A. Kusari, K. A. Kenner, F. Liu, J. Chernoff, T. A. Gustafson, and J. Kusari. 1997. Protein-Tyrosine Phosphatase 1B Complexes with the Insulin Receptor in Vivo and Is Tyrosine-phosphorylated in the Presence of Insulin. J. Biol. Chem. 272:1639–1645.

Bardelli, A., P. Longati, T. A. Williams, S. Benvenuti, and P. M. Comoglio. 1999. A Peptide Representing the Carboxyl-terminal Tail of the Met Receptor Inhibits Kinase Activity and Invasive Growth. J. Biol. Chem. 274:29274–29281.

Bertero, T., C. Gastaldi, I. Bourget-Ponzio, B. Mari, G. Meneguzzi, P. Barbry, G. Ponzio, and R. Rezzonico. 2013. CDC25A targeting by miR-483-3p decreases CCND–CDK4/6 assembly and contributes to cell cycle arrest. Cell Death Differ. 20:800–811.

Cao, C., Y. Leng, and D. Kufe. 2003. Catalase Activity Is Regulated by c-Abl and Arg in the Oxidative Stress Response. J. Biol. Chem. 278:29667–29675.

Dar, A. C., T. E. Dever, and F. Sicheri. 2005. Higher-Order Substrate Recognition of eIF2α by the RNA-Dependent Protein Kinase PKR. Cell 122:887–900.

Dougherty, M. K., J. Müller, D. A. Ritt, M. Zhou, X. Z. Zhou, T. D. Copeland, T. P. Conrads, T. D. Veenstra, K. P. Lu, and D. K. Morrison. 2005. Regulation of Raf-1 by Direct Feedback Phosphorylation. Mol. Cell 17:215–224.

Dérijard, B., M. Hibi, I.-H. Wu, T. Barrett, B. Su, T. Deng, M. Karin, and R. J. Davis. 1994. JNK1: A protein kinase stimulated by UV light and Ha-Ras that binds and phosphorylates the c-Jun activation domain. Cell 76:1025–1037.

Ellerbroek, S. M., K. Wennerberg, and K. Burridge. 2003. Serine Phosphorylation Negatively Regulates RhoA in Vivo. J. Biol. Chem. 278:19023–19031.

Feng, W., X. Duan, J. Liu, J. Xiao, and R. E. Brown. 2008. Morphoproteomic Evidence of Constitutively Activated and Overexpressed mTOR Pathway in Cervical Squamous Carcinoma and High Grade Squamous Intraepithelial Lesions. Int. J. Clin. Exp. Pathol. 2:249–260.

Filipp, D., J. Zhang, B. L. Leung, A. Shaw, S. D. Levin, A. Veillette, and M. Julius. 2003. Regulation of Fyn Through Translocation of Activated Lck into Lipid Rafts. J. Exp. Med. 197:1221–1227.

Floyd, Z. E., and J. M. Stephens. 2002. Interferon-γ-mediated Activation and Ubiquitin-Proteasome-dependent Degradation of PPARγ in Adipocytes. J. Biol. Chem. 277:4062–4068.

Frey, J. W., B. L. Jacobs, C. A. Goodman, and T. A. Hornberger. 2014. A Role for Raptor Phosphorylation in the Mechanical Activation of mTOR Signaling. Cell. Signal. 26:313–322.

Fu, C., C. W. Turck, T. Kurosaki, and A. C. Chan. 1998. BLNK: a Central Linker Protein in B Cell Activation. Immunity 9:93–103.

Furdui, C. M., E. D. Lew, J. Schlessinger, and K. S. Anderson. 2006. Autophosphorylation of FGFR1 Kinase Is Mediated by a Sequential and Precisely Ordered Reaction. Mol. Cell 21:711–717.

Gong, Q., X. Jin, A. M. Akk, N. Foger, M. White, G. Gong, J. B. Wardenburg, and A. C. Chan. 2001. Requirement for Tyrosine Residues 315 and 319 within ζ Chain–Associated Protein 70 for T Cell Development. J. Exp. Med. 194:507–518.

Gururaj, A., C. J. Barnes, R. K. Vadlamudi, and R. Kumar. 2004. Regulation of phosphoglucomutase 1 phosphorylation and activity by a signaling kinase. Oncogene 23:8118–8127.

Hanaka, H., T. Shimizu, and T. Izumi. 2005. Stress-induced nuclear export of 5-lipoxygenase. Biochem. Biophys. Res. Commun. 338:111–116.

Hausel, P., H. Latado, F. Courjault-Gautier, and E. Felley-Bosco. 2006. Src-mediated phosphorylation regulates subcellular distribution and activity of human inducible nitric oxide synthase. Oncogene 25:198.

Hunkeler, M., A. Hagmann, E. Stuttfeld, M. Chami, Y. Guri, H. Stahlberg, and T. Maier. 2018. Structural basis for regulation of human acetyl-CoA carboxylase. Nature 558:470–474.

Inoki, K., Y. Li, T. Zhu, J. Wu, and K.-L. Guan. 2002. TSC2 is phosphorylated and inhibited by Akt and suppresses mTOR signalling. Nat. Cell Biol. 4:648–657.

Ivison, S. M., M. A. S. Khan, N. R. Graham, C. Q. Bernales, A. Kaleem, C. O. Tirling, A. Cherkasov, and T. S. Steiner. 2007. A phosphorylation site in the Toll-like receptor 5 TIR domain is required for inflammatory signalling in response to flagellin. Biochem. Biophys. Res. Commun. 352:936–941.

Jurek, A., K. Amagasaki, A. Gembarska, C.-H. Heldin, and J. Lennartsson. 2009. Negative and Positive Regulation of MAPK Phosphatase 3 Controls Platelet-derived Growth Factor-induced Erk Activation. J. Biol. Chem. 284:4626–4634.

Kebache, S., E. Cardin, D. T. Nguyên, E. Chevet, and L. Larose. 2004. Nck-1 Antagonizes the Endoplasmic Reticulum Stress-induced Inhibition of Translation. J. Biol. Chem. 279:9662–9671.

Kim, Y. J., F. Sekiya, B. Poulin, Y. S. Bae, and S. G. Rhee. 2004. Mechanism of B-Cell Receptor-Induced Phosphorylation and Activation of Phospholipase C-γ2. Mol. Cell. Biol. 24:9986–9999.

Kinoshita, T., M. Shirouzu, A. Kamiya, K. Hashimoto, S. Yokoyama, and A. Miyajima. 1997. Raf/MAPK and rapamycin-sensitive pathways mediate the anti-apoptotic function of p21Ras in IL-3-dependent hematopoietic cells. Oncogene 15:619–627.

Koul, D., S. A. Jasser, Y. Lu, M. A. Davies, R. Shen, Y. Shi, G. B. Mills, and W. A. Yung. 2002. Motif analysis of the tumor suppressor gene MMAC/PTEN identifies tyrosines critical for tumor suppression and lipid phosphatase activity. Oncogene 21:2357–2364.

Kumar, K. G. S., J. J. Krolewski, and S. Y. Fuchs. 2004. Phosphorylation and Specific Ubiquitin Acceptor Sites Are Required for Ubiquitination and Degradation of the IFNAR1 Subunit of Type I Interferon Receptor. J. Biol. Chem. 279:46614–46620.

Kurokawa, M., C. Zhao, T. Reya, and S. Kornbluth. 2008. Inhibition of Apoptosome Formation by Suppression of Hsp90β Phosphorylation in Tyrosine Kinase-Induced Leukemias. Mol. Cell. Biol. 28:5494–5506.

Kwon, T., D. Y. Kwon, J. Chun, J. H. Kim, and S. S. Kang. 2000. Akt Protein Kinase Inhibits Rac1-GTP Binding through Phosphorylation at Serine 71 of Rac1. J. Biol. Chem. 275:423–428.

Lang, V., J. Janzen, G. Z. Fischer, Y. Soneji, S. Beinke, A. Salmeron, H. Allen, R. T. Hay, Y. Ben-Neriah, and S. C. Ley. 2003. βTrCP-Mediated Proteolysis of NF-κB1 p105 Requires Phosphorylation of p105 Serines 927 and 932. Mol. Cell. Biol. 23:402–413.

Langer, T., S. Sreeramulu, M. Vogtherr, B. Elshorst, M. Betz, U. Schieborr, K. Saxena, and H. Schwalbe. 2005. Folding and activity of cAMP-dependent protein kinase mutants. FEBS Lett. 579:4049–4054.

Lekmine, F., A. Sassano, S. Uddin, J. Smith, B. Majchrzak, S. M. Brachmann, N. Hay, E. N. Fish, and L. C. Platanias. 2004. Interferon-γ engages the p70 S6 kinase to regulate phosphorylation of the 40S S6 ribosomal protein. Exp. Cell Res. 295:173–182.

Li, S., A. D. Couvillon, B. B. Brasher, and R. A. Van Etten. 2001. Tyrosine phosphorylation of Grb2 by Bcr/Abl and epidermal growth factor receptor: a novel regulatory mechanism for tyrosine kinase signaling. EMBO J. 20:6793–6804.

Li, X., M. Yang, Z. Yu, S. Tang, L. Wang, X. Cao, and T. Chen. 2017. The tyrosine kinase Src promotes phosphorylation of the kinase TBK1 to facilitate type I interferon production after viral infection. Sci. Signal. 10.

Lu, M., and J. Y.-J. Shyy. 2006. Sterol regulatory element-binding protein 1 is negatively modulated by PKA phosphorylation. Am. J. Physiol.-Cell Physiol. 290:C1477–C1486.

MacNicol, M. C., A. J. Muslin, and A. M. MacNicol. 2000. Disruption of the 14-3-3 Binding Site within the B-Raf Kinase Domain Uncouples Catalytic Activity from PC12 Cell Differentiation. J. Biol. Chem. 275:3803–3809.

Mahajan, S., A. Vassilev, N. Sun, Z. Ozer, C. Mao, and F. M. Uckun. 2001. Transcription Factor STAT5A Is a Substrate of Bruton's Tyrosine Kinase in B Cells. J. Biol. Chem. 276:31216–31228.

Mustelin, T., T. Pessa-Morikawa, M. Autero, M. Gassmann, L. C. Andersson, C. G. Gahmberg, and P. Burn. 1992. Regulation of the p59fyn protein tyrosine kinase by the CD45 phosphotyrosine phosphatase. Eur. J. Immunol. 22:1173–1178.

Nakatani, K., H. Sakaue, D. A. Thompson, R. J. Weigel, and R. A. Roth. 1999. Identification of a Human Akt3 (Protein Kinase B γ) Which Contains the Regulatory Serine Phosphorylation Site. Biochem. Biophys. Res. Commun. 257:906–910.

Nasrin, N., V. K. Kaushik, E. Fortier, D. Wall, K. J. Pearson, R. de Cabo, and L. Bordone. 2009. JNK1 Phosphorylates SIRT1 and Promotes Its Enzymatic Activity. PLoS ONE 4 Available at https://www.ncbi.nlm.nih.gov/pmc/articles/PMC2793009/ (verified 16 April 2020).

Ong, S. T., M. Freeley, J. Skubis-Zegadło, M. H. U. T. Fazil, D. Kelleher, F. Fresser, G. Baier, N. K. Verma, and A. Long. 2014. Phosphorylation of Rab5a Protein by Protein Kinase Cϵ Is Crucial for T-cell Migration. J. Biol. Chem. 289:19420–19434.

Park, H.-S., M.-S. Kim, S.-H. Huh, J. Park, J. Chung, S. S. Kang, and E.-J. Choi. 2002. Akt (Protein Kinase B) Negatively Regulates SEK1 by Means of Protein Phosphorylation. J. Biol. Chem. 277:2573–2578.

Peck, D., and C. M. Isacke. 1998. Hyaluronan-dependent cell migration can be blocked by a CD44 cytoplasmic domain peptide containing a phosphoserine at position 325. J. Cell Sci. 111:1595–1601.

Perino, A., A. Ghigo, E. Ferrero, F. Morello, G. Santulli, G. S. Baillie, F. Damilano, A. J. Dunlop, C. Pawson, R. Walser, R. Levi, F. Altruda, L. Silengo, L. K. Langeberg, G. Neubauer, S. Heymans, G. Lembo, M. P. Wymann, R. Wetzker, M. D. Houslay, G. Iaccarino, J. D. Scott, and E. Hirsch. 2011. Integrating Cardiac PIP3 and cAMP Signaling through a PKA Anchoring Function of p110γ. Mol. Cell 42:84–95.

Procaccia, S., M. Ordan, I. Cohen, S. Bendetz-Nezer, and R. Seger. 2017. Direct binding of MEK1 and MEK2 to AKT induces Foxo1 phosphorylation, cellular migration and metastasis. Sci. Rep. 7 Available at https://www.ncbi.nlm.nih.gov/pmc/articles/PMC5320536/ (verified 4 February 2020).

Puigserver, P., J. Rhee, J. Lin, Z. Wu, J. C. Yoon, C.-Y. Zhang, S. Krauss, V. K. Mootha, B. B. Lowell, and B. M. Spiegelman. 2001. Cytokine Stimulation of Energy Expenditure through p38 MAP Kinase Activation of PPARγ Coactivator-1. Mol. Cell 8:971–982.

Puustinen, P., A. Keldsbo, E. Corcelle-Termeau, K. Ngoei, S. L. Sønder, T. Farkas, K. K. Andersen, J. S. Oakhill, and M. Jäättelä. 2020. DNA-dependent protein kinase regulates lysosomal AMP-dependent protein kinase activation and autophagy. Autophagy 0:1–18.

Qasimi, P., A. Ming-Lum, A. Ghanipour, C. J. Ong, M. E. Cox, J. Ihle, N. Cacalano, A. Yoshimura, and A. L.-F. Mui. 2006. Divergent Mechanisms Utilized by SOCS3 to Mediate Interleukin-10 Inhibition of Tumor Necrosis Factor α and Nitric Oxide Production by Macrophages. J. Biol. Chem. 281:6316–6324.

Raingeaud, J., A. J. Whitmarsh, T. Barrett, B. Dérijard, and R. J. Davis. 1996. MKK3- and MKK6-regulated gene expression is mediated by the p38 mitogen-activated protein kinase signal transduction pathway. Mol. Cell. Biol. 16:1247–1255.

Rohde, C. M., J. Schrum, and A. W.-M. Lee. 2004. A Juxtamembrane Tyrosine in the Colony Stimulating Factor-1 Receptor Regulates Ligand-induced Src Association, Receptor Kinase Function, and Down-regulation. J. Biol. Chem. 279:43448–43461.

Roskoski, R. 2017. ROS1 protein-tyrosine kinase inhibitors in the treatment of ROS1 fusion protein-driven non-small cell lung cancers. Pharmacol. Res. 121:202–212.

Salameh, A., F. Galvagni, M. Bardelli, F. Bussolino, and S. Oliviero. 2005. Direct recruitment of CRK and GRB2 to VEGFR-3 induces proliferation, migration, and survival of endothelial cells through the activation of ERK, AKT, and JNK pathways. Blood 106:3423–3431.

Sathyanarayana, P., A. Dev, A. Pradeep, M. Ufkin, J. D. Licht, and D. M. Wojchowski. 2012. Spry1 as a novel regulator of erythropoiesis, EPO/EPOR target, and suppressor of JAK2. Blood 119:5522–5531.

Schumacher, A. M., J. P. Schavocky, A. V. Velentza, S. Mirzoeva, and D. M. Watterson. 2004. A Calmodulin-Regulated Protein Kinase Linked to Neuron Survival Is a Substrate for the Calmodulin-Regulated Death-Associated Protein Kinase. Biochemistry 43:8116–8124.

Schuringa, J. J., L. J. Jonk, W. H. Dokter, E. Vellenga, and W. Kruijer. 2000. Interleukin-6-induced STAT3 transactivation and Ser727 phosphorylation involves Vav, Rac-1 and the kinase SEK-1/MKK-4 as signal transduction components. Biochem. J. 347:89–96.

Shinde, S. R., and S. Maddika. 2016. PTEN modulates EGFR late endocytic trafficking and degradation by dephosphorylating Rab7. Nat. Commun. 7 Available at https://www.ncbi.nlm.nih.gov/pmc/articles/PMC4754336/ (verified 22 April 2020).

Shiraha, H., A. Glading, J. Chou, Z. Jia, and A. Wells. 2002. Activation of m-Calpain (Calpain II) by Epidermal Growth Factor Is Limited by Protein Kinase A Phosphorylation of m-Calpain. Mol. Cell. Biol. 22:2716–2727.

Singhirunnusorn, P., S. Suzuki, N. Kawasaki, I. Saiki, and H. Sakurai. 2005. Critical Roles of Threonine 187 Phosphorylation in Cellular Stress-induced Rapid and Transient Activation of Transforming Growth Factor-β-activated Kinase 1 (TAK1) in a Signaling Complex Containing TAK1-binding Protein TAB1 and TAB2. J. Biol. Chem. 280:7359–7368.

Soliman, G. A., H. A. Acosta-Jaquez, E. A. Dunlop, B. Ekim, N. E. Maj, A. R. Tee, and D. C. Fingar. 2010. mTOR Ser-2481 Autophosphorylation Monitors mTORC-specific Catalytic Activity and Clarifies Rapamycin Mechanism of Action. J. Biol. Chem. 285:7866–7879.

Song, T. L., M.-L. Nairismägi, Y. Laurensia, J.-Q. Lim, J. Tan, Z.-M. Li, W.-L. Pang, A. Kizhakeyil, G.-C. Wijaya, D.-C. Huang, S. Nagarajan, B. K.-H. Chia, D. Cheah, Y.-H. Liu, F. Zhang, H.-L. Rao, T. Tang, E. K.-Y. Wong, J.-X. Bei, J. Iqbal, N.-F. Grigoropoulos, S.-B. Ng, W.-J. Chng, B.-T. Teh, S.-Y. Tan, N. K. Verma, H. Fan, S.-T. Lim, and C.-K. Ong. 2018. Oncogenic activation of the STAT3 pathway drives PD-L1 expression in natural killer/T-cell lymphoma. Blood 132:1146–1158.

Song, L., P. D. Sarno, and R. S. Jope. 2002. Central Role of Glycogen Synthase Kinase-3β in Endoplasmic Reticulum Stress-induced Caspase-3 Activation. J. Biol. Chem. 277:44701–44708.

Sriram, G., W. Jankowski, C. Kasikara, C. Reichman, T. Saleh, K.-Q. Nguyen, J. Li, P. Hornbeck, K. Machida, T. Liu, H. Li, C. G. Kalodimos, and R. B. Birge. 2015. Iterative Tyrosine Phosphorylation Controls Non-canonical Domain Utilization in Crk. Oncogene 34:4260–4269.

Stephens, R. M., D. M. Loeb, T. D. Copeland, T. Pawson, L. A. Greene, and D. R. Kaplan. 1994. Trk receptors use redundant signal transduction pathways involving SHC and PLC-γ1 to mediate NGF responses. Neuron 12:691–705.

Taha-abdelaziz, K., T. N. Alkie, D. C. Hodgins, B. Shojadoost, and S. Sharif. 2016. Characterization of host responses induced by Toll-like receptor ligands in chicken cecal tonsil cells. Vet. Immunol. Immunopathol. 174:19–25.

Taylor, D. R., B. Tian, P. R. Romano, A. G. Hinnebusch, M. M. C. Lai, and M. B. Mathews. 2001. Hepatitis C Virus Envelope Protein E2 Does Not Inhibit PKR by Simple Competition with Autophosphorylation Sites in the RNA-Binding Domain. J. Virol. 75:1265–1273.

Thien, C. B. F., S. A. Dagger, J. H. Steer, F. Koentgen, E. S. Jansen, C. L. Scott, and W. Y. Langdon. 2010. c-Cbl Promotes T Cell Receptor-induced Thymocyte Apoptosis by Activating the Phosphatidylinositol 3-Kinase/Akt Pathway. J. Biol. Chem. 285:10969–10981.

Thuille, N., I. Heit, F. Fresser, N. Krumböck, B. Bauer, S. Leuthaeusser, S. Dammeier, C. Graham, T. D. Copeland, S. Shaw, and G. Baier. 2005. Critical role of novel Thr-219 autophosphorylation for the cellular function of PKCθ in T lymphocytes. EMBO J. 24:3869–3880.

Tsang, E., A. M. Giannetti, D. Shaw, M. Dinh, J. K. Y. Tse, S. Gandhi, H. Ho, S. Wang, E. Papp, and J. M. Bradshaw. 2008. Molecular Mechanism of the Syk Activation Switch. J. Biol. Chem. 283:32650–32659.

Vanhaesebroeck, B., K. Higashi, C. Raven, M. Welham, S. Anderson, P. Brennan, S. G. Ward, and M. D. Waterfield. 1999. Autophosphorylation of p110delta phosphoinositide 3-kinase: a new paradigm for the regulation of lipid kinases in vitro and in vivo. EMBO J. 18:1292–1302.

Venkitaraman, A. R., and R. J. Cowling. 1994. Interleukin-7 induces the association of phosphatidylinositol 3-kinase with the α chain of the interleukin-7 receptor. Eur. J. Immunol. 24:2168–2174.

Wang, R., P. R. Griffin, E. C. Small, and J. E. Thompson. 2003. Mechanism of Janus kinase 3-catalyzed phosphorylation of a Janus kinase 1 activation loop peptide. Arch. Biochem. Biophys. 410:7–15.

Wang, A. H., M. J. Kruhlak, J. Wu, N. R. Bertos, M. Vezmar, B. I. Posner, D. P. Bazett-Jones, and X.-J. Yang. 2000. Regulation of Histone Deacetylase 4 by Binding of 14-3-3 Proteins. Mol. Cell. Biol. 20:6904–6912.

Warden, S. M., C. Richardson, J. O'Donnell, D. Stapleton, B. E. Kemp, and L. A. Witters. 2001. Post-translational modifications of the beta-1 subunit of AMP-activated protein kinase affect enzyme activity and cellular localization. Biochem. J. 354:275–283.

Wieser, R., J. L. Wrana, and J. Massagué. 1995. GS domain mutations that constitutively activate T beta R-I, the downstream signaling component in the TGF-beta receptor complex. EMBO J. 14:2199–2208.

Won, K. A., and S. I. Reed. 1996. Activation of cyclin E/CDK2 is coupled to site-specific autophosphorylation and ubiquitin-dependent degradation of cyclin E. EMBO J. 15:4182–4193.

Wood, P., V. Mulay, M. Darabi, K. C. Chan, J. Heeren, A. Pol, G. Lambert, K.-A. Rye, C. Enrich, and T. Grewal. 2011. Ras/Mitogen-activated Protein Kinase (MAPK) Signaling Modulates Protein Stability and Cell Surface Expression of Scavenger Receptor SR-BI. J. Biol. Chem. 286:23077–23092.

Xiao, G., E. W. Harhaj, and S.-C. Sun. 2001. NF-κB-Inducing Kinase Regulates the Processing of NF-κB2 p100. Mol. Cell 7:401–409.

Yang, B. S., C. A. Hauser, G. Henkel, M. S. Colman, C. Van Beveren, K. J. Stacey, D. A. Hume, R. A. Maki, and M. C. Ostrowski. 1996. Ras-mediated phosphorylation of a conserved threonine residue enhances the transactivation activities of c-Ets1 and c-Ets2. Mol. Cell. Biol. 16:538–547.

Yang, C.-Y., J.-P. Li, L.-L. Chiu, J.-L. Lan, D.-Y. Chen, H.-C. Chuang, C.-Y. Huang, and T.-H. Tan. 2014. Dual-Specificity Phosphatase 14 (DUSP14/MKP6) Negatively Regulates TCR Signaling by Inhibiting TAB1 Activation. J. Immunol. 192:1547–1557.

Yang, G., D. S. Murashige, S. J. Humphrey, and D. E. James. 2015. A Positive Feedback Loop between Akt and mTORC2 via SIN1 Phosphorylation. Cell Rep. 12:937–943.

Yokoyama, N., N. C. Reich, and W. T. Miller. 2001. Involvement of Protein Phosphatase 2A in the Interleukin-3-Stimulated Jak2-Stat5 Signaling Pathway. J. Interferon Cytokine Res. 21:369–378.

Yugi, K., H. Kubota, Y. Toyoshima, R. Noguchi, K. Kawata, Y. Komori, S. Uda, K. Kunida, Y. Tomizawa, Y. Funato, H. Miki, M. Matsumoto, K. I. Nakayama, K. Kashikura, K. Endo, K.

Ikeda, T. Soga, and S. Kuroda. 2014. Reconstruction of Insulin Signal Flow from Phosphoproteome and Metabolome Data. Cell Rep. 8:1171–1183.

Zhan, Q., Q. Ge, T. Ohira, T. V. Dyke, and J. A. Badwey. 2003. p21-Activated Kinase 2 in Neutrophils Can Be Regulated by Phosphorylation at Multiple Sites and by a Variety of Protein Phosphatases. J. Immunol. 171:3785–3793.

Zhang, J., H. Feng, J. Zhao, E. R. Feldman, S.-Y. Chen, W. Yuan, C. Huang, O. Akbari, S. A. Tibbetts, and P. Feng. 2016. IκB Kinase ε Is an NFATc1 Kinase that Inhibits T Cell Immune Response. Cell Rep. 16:405–418.

Zhang, D., M. Guo, W. Zhang, and X.-Y. Lu. 2011. Adiponectin Stimulates Proliferation of Adult Hippocampal Neural Stem/Progenitor Cells through Activation of p38 Mitogen-activated Protein Kinase (p38MAPK)/Glycogen Synthase Kinase 3β (GSK-3β)/β-Catenin Signaling Cascade. J. Biol. Chem. 286:44913–44920.

Zhang, Y., Y.-J. Ren, L.-C. Guo, C. Ji, J. Hu, H.-H. Zhang, Q.-H. Xu, W.-D. Zhu, Z.-J. Ming, Y.-S. Yuan, X. Ren, J. Song, and J.-M. Yang. 2017. Nucleus accumbens-associated protein-1 promotes glycolysis and survival of hypoxic tumor cells via the HDAC4-HIF-1α axis. Oncogene 36:4171–4181.

Zhou, J., Z. Shao, R. Kerkela, H. Ichijo, A. J. Muslin, C. Pombo, and T. Force. 2009. Serine 58 of 14-3-3ζ Is a Molecular Switch Regulating ASK1 and Oxidant Stress-Induced Cell Death. Mol. Cell. Biol. 29:4167–4176.
