# Supplementary material for: Modern broiler chickens exhibit a differential gastrointestinal immune and metabolic response to repeated CpG injection relative to a 1950s heritage broiler breed
Source: Front Physiol. 2024 Nov 1;15:1473202. doi: 10.3389/fphys.2024.1473202 (PMC11565619; doi:10.3389/fphys.2024.1473202)
Supplement: Supplementary file 6 [file Image1.pdf]

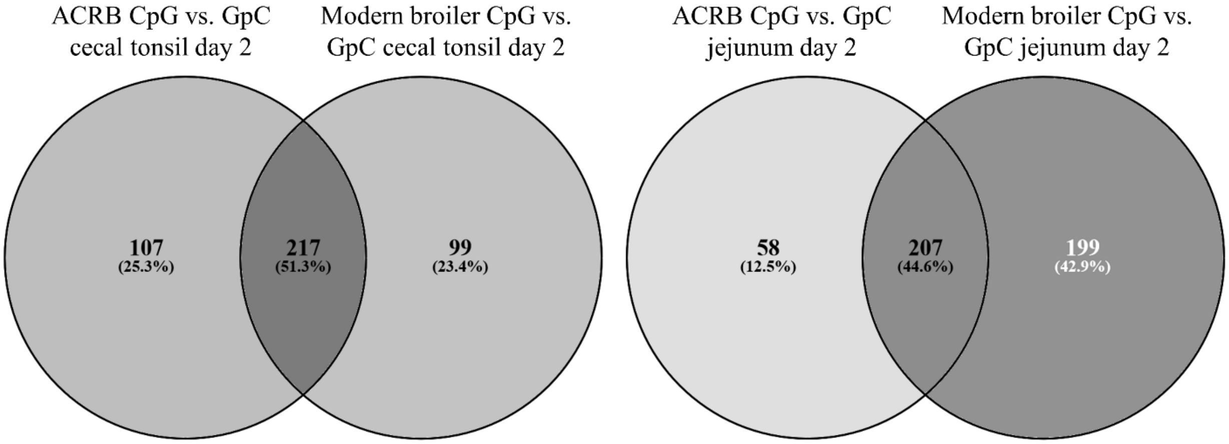

Supplementary Figure 1: Significant peptides in the treatment birds' samples compared to the control birds' samples were used to generate these Venn diagrams using the program Venny (https://bioinfogp.cnb.csic.es/tools/venny/). The Uniprot IDs for each significant peptide in each bird type were compared to identify significant peptides in common between the two bird types and unique between the two bird types. This comparison did not take into account differences in the direction of the change in phosphorylation, whether increased or decreased.

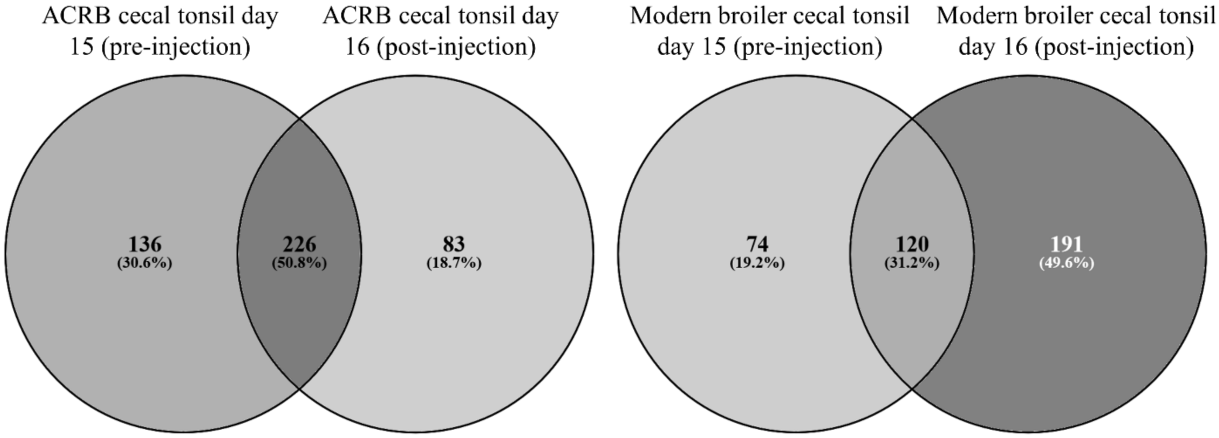

Supplementary Figure 2: These Venn diagrams were generated using Venny (https://bioinfogp.cnb.csic.es/tools/venny/) to compare the list of significant peptides in the day 15 cecal tonsil to the list of significant peptides in the day 16 cecal tonsils for both bird types (left ACRB birds, right modern broiler birds).

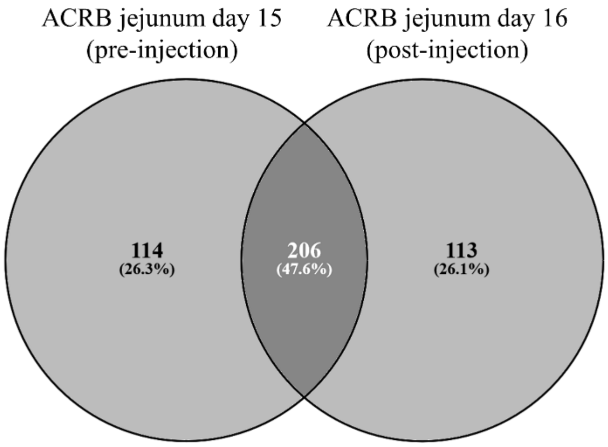

Supplementary Figure 3: These Venn diagrams were generated using Venny (https://bioinfogp.cnb.csic.es/tools/venny/) to compare the list of significant peptides in the day 15 jejunum to the list of significant peptides in the day 16 jejunum for both bird types (left ACRB birds, right modern broiler birds).

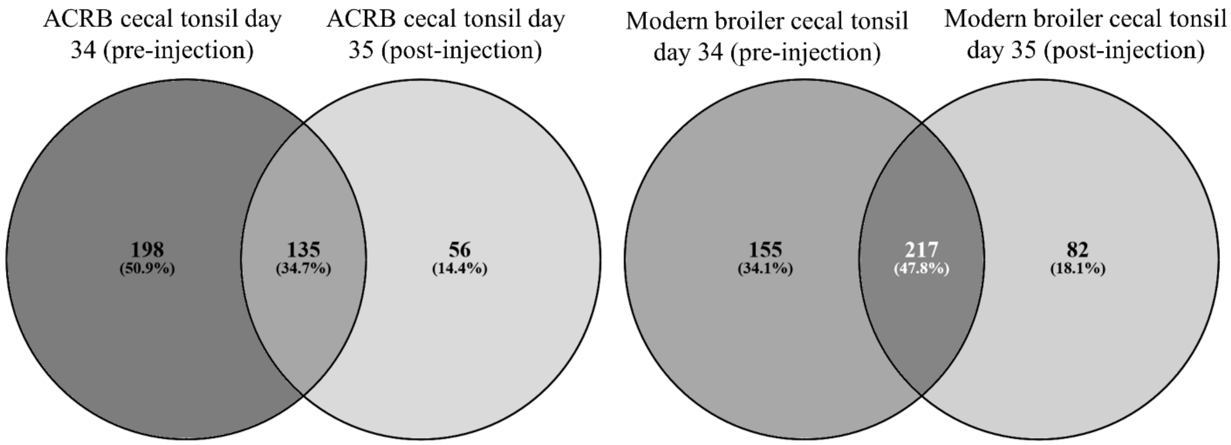

Supplementary Figure 4: These Venn diagrams were generated using Venny (https://bioinfogp.cnb.csic.es/tools/venny/) to compare the list of significant peptides in the day 34 cecal tonsil to the list of significant peptides in the day 35 cecal tonsils for both bird types (left ACRB birds, right modern broiler birds).

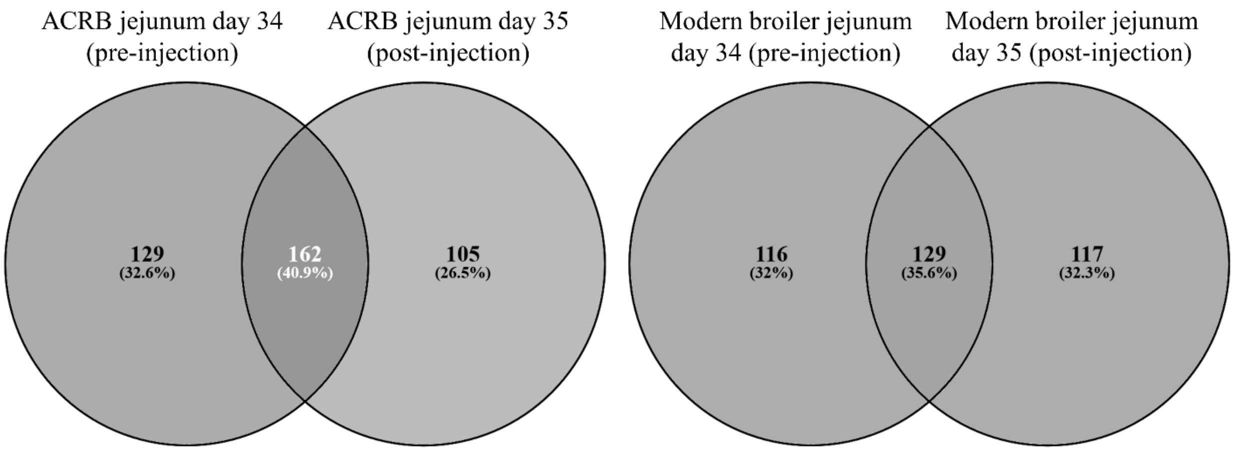

Supplementary Figure 5: These Venn diagrams were generated using Venny (https://bioinfogp.cnb.csic.es/tools/venny/) to compare the list of significant peptides in the day 34 jejunum to the list of significant peptides in the day 35 jejunum for both bird types (left ACRB birds, right modern broiler birds).
